# Trogopterins A–C: Three new neolignans from feces of *Trogopterus xanthipes*

**DOI:** 10.3762/bjoc.10.313

**Published:** 2014-12-11

**Authors:** Soyoon Baek, Xuikui Xia, Byung Sun Min, Chanil Park, Sang Hee Shim

**Affiliations:** 1 School of Biotechnology, Yeungnam University, Daedong, Gyeongsan, Gyeongbuk 712-749, South Korea; 2 Key Laboratory for Applied Microbiology of Shandong Province, Biotechnology Center of Shandong Academy of Sciences, Jinan 250014, P. R. China; 3 College of Pharmacy, Catholic University of Daegu, 13-13 Hayang-ro, Gyeongsan, Gyeongbuk 712-702, South Korea; 4 Department of Marine Biology & Aquaculture, Gyeongsang National University, Tongyeong, Gyeongnam 650-160, South Korea

**Keywords:** cytotoxic activity, neolignans, *Trogopterus xanthipes*

## Abstract

Seven compounds, including three neolignans **1**–**3**, a norlignan **4**, and three diterpenoids **5**–**7**, were isolated from the feces of *Trogopterus xanthipes*. Structures of these compounds were identified by 1D and 2D NMR as well as MS. The absolute configurations of compounds **1**, **2**, and **4** were determined by comparing CD spectra and optical rotations. Among the isolated compounds, **1**–**3** were novel and subsequently named trogopterins A, B, and C, respectively. Likewise, compound **4** was isolated from nature for the first time. Cytotoxic activities of compounds **1**–**4** were evaluated. Compounds **1**–**3** exhibited moderate cytotoxic activities against HL-60 cells with IC_50_ values of 34.77–45.68 μM.

## Introduction

Chemical studies of natural products including ones derived from plants and microorganisms have led to the isolation of numerous novel metabolites with biological activities [1–2]. As a continuation of these investigations, feces from *Trogopterus* were selected since organic extracts from this material were found to exhibit potent cytotoxic activities in our preliminary study. *Trogopterus* feces, called “Wulingzhi”, are dried excrements of *Trogopterus* (*T.*) *xanthipes* Milne-Edwars (Petauristidae) that are known as complex-toothed flying squirrels to eat branches, leaves, and fruits of pine trees [3]. *Trogopterus* feces have been reported to promote blood circulation and resolve stasis. Thus, this material has been used as traditional medicine for treating amenorrhea, dysmenorrheal, menstrual pain, and retained lochia due to stasis [4].

Recent studies have indicated that *Trogopterus* feces mainly consist of terpenoids [5–9], phenolic acids, sterols, aliphatics, fatty acids, and flavonoids [10–11]. They have been reported to possess various pharmacological properties such as controlling of antithrombin levels, inhibition of platelet aggregation, cytotoxic activity, immunity enhancement, and anti-inflammatory activities. Isolation of compounds from the methanol extract of *Trogopterus* feces was presented before by our group [12]. In the present investigation, chemical evaluation of fecal extracts led to the isolation of seven compounds including three new neolignans named trogopterins A–C (**1–3**). Here we describe the isolation, structure, and cytotoxic activities of compounds **1–3**.

## Results and Discussion

**Figure 1:**
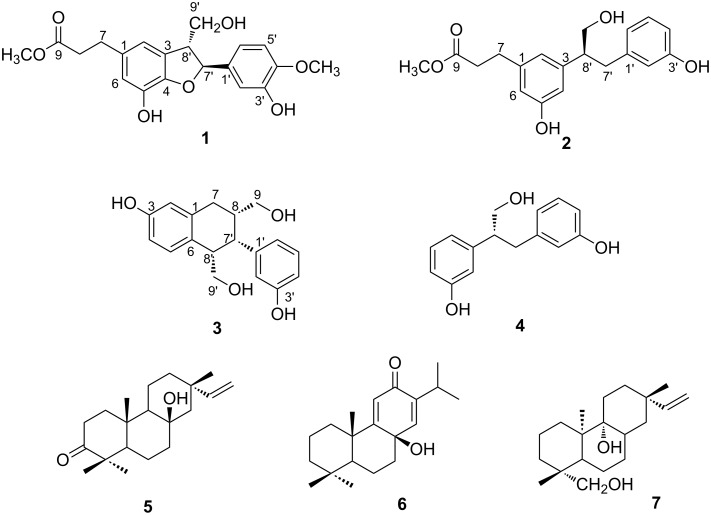
Structures of compounds **1–7** isolated from feces of *Trogopterus*.

Seven compounds, including three neolignans (**1**–**3**), a phenolic compound **4**, and three diterpenoids (**5**–**7**), were isolated from the methanolic extract of *T. xanthipes* feces. Their chemical names are methyl 3-((2*S*,3*R*)-7-hydroxy-2-(3-hydroxy-4-methoxyphenyl)-3-(hydroxymethyl)-2,3-dihydrobenzofuran-5-yl)propanoate (**1**), (*S*)-methyl 3-(3-hydroxy-5-(1-hydroxy-3-(3-hydroxyphenyl)propan-2-yl)phenyl)propanoate (**2**), ((1*RS*,2*RS*,3*RS*)-6-hydroxy-2-(3-hydroxyphenyl)-1,2,3,4-tetrahydronaphthalene-1,3-diyl)dimethanol (**3**), ((*R*)-3,3'-(3-hydroxypropane-1,2-diyl)diphenol (**4**) [13], 8β-hydroxy-3-oxopimara-15-ene (**5**) [14], 9β-hydroxy-9(11),13-abietadien-12-one (**6**) [15], and *ent*-pimar-15-en-9α,19-diol (**7**) [16] based on spectral data (Figure 1). Compounds **1**–**3** were identified to be new and compound **4** was isolated from nature for the first time. In addition, compounds **6** and **7** were reported from this species in this study for the first time.

Compound **1** was isolated as colorless oil. The molecular formula of compound **1** was determined to be C_20_H_22_O_7_ (ten unsaturations) based on ^1^H, ^13^C, and HMQC data, and verified by HREIMS (Table 1 and Table 2). Nineteen protons were bound to carbons, so three exchangeable hydrogens were present. Detailed analysis of the ^1^H, ^13^C, and HMQC spectra of compound **1** revealed the presence of two methoxy groups at δ_H_ 3.63 (δ_C_ 52.0) and δ_H_ 3.80 (δ_C_ 56.4), an oxygenated methine group at δ_H_ 5.47 (δ_C_ 88.8), an oxygenated methylene group at δ_H_ 3.80 and 3.73 (δ_C_ 65.1), an sp^3^ methine group at δ_H_ 3.43 (δ_C_ 55.7), two sp^3^ methylene groups at δ_H_ 2.78 (δ_C_ 31.7) and δ_H_ 2.56 (δ_C_ 37.1); and five sp^2^ methine groups at δ_H_ 6.95 (δ_C_ 110.5), δ_H_ 6.82 (δ_C_ 119.7), δ_H_ 6.74 (δ_C_ 116.1), δ_H_ 6.59 (δ_C_ 116.5), and δ_H_ 6.55 (δ_C_ 116.9). In addition, seven sp^2^ non-hydrogenated carbon signals appeared at δ_C_ 149.1, 147.4, 146.9, 142.0, 135.2, 135.0, and 130.0 along with an ester at 175.3. Based on ^1^H and ^13^C NMR findings (Table 1 and Table 2), compound **1** was hypothesized to have a lignan skeleton formed through oxidative coupling of two phenylpropanoids units.

**Table 1:** ^1^H NMR data for compounds **1**–**4** (δ, ppm, and coupling constant *J* in Hz).

Position	**1**^a^	**2**^b^	**3**^a^	**4**^a^

1				
2	6.59 (s)	6.88 (br s)		6.61 (t, 3.0)
3			6.51 (d, 8.4)	
4		7.19 (br s)	6.43 (dd, 8.4, 2.4)	6.58 (ddd, 7.8, 3.0, 0.6)
5				7.05 (t, 7.8)
6	6.55 (s)	6.98 (br s)	6.54 (br s)	6.63 (d, 7.8)
7	2.78 (t, 7.8)	2.93 (t, 8.4)	2.76 (d, 7.8)	2.92 (m)
8a	2.56 (t, 7.8)	2.64 (t, 8.4)	1.98 (m)	3.68 (dd, 6.6, 1.2)
8b				3.64 (m)
9a			3.66 (dd, 11, 3.6)	2.71 (dd, 13, 8.4)
9b			3.61 (dd, 11, 6.0)	3.00 (dd, 13,6.6)
9-OCH_3_	3.63 (s)	3.57 (s)		
1'				
2'	6.95 (s)	7.20 (d, 1.8)	6.55 (br s)	6.51 (s)
3'				
4'		7.01 (dd, 7.8, 1.8)	6.62 (dd, 7.8, 2.4)	6.52 (d, 7.2)
5'	6.74 (d, 8.4)	7.20 (t, 7.8)	7.09 (t, 7.8)	6.97 (t, 7.2)
6'	6.82 (br d, 8.4)	6.90 (d, 7.2)	6.64 (br d, 7.8)	6.53 (d, 7.2)
7'a	5.47 (d, 6.0)	3.47 (dd, 13, 6.0)	3.84 (d, 10)	
7'b		3.12 (dd, 13, 8.4)		
8'	3.43 (m)	3.40 (m)	1.79 (m)	
9'a	3.80 (m)	4.14 (m)	3.39 (dd, 10, 4.2)	
9'b	3.73 (dd, 11, 7.2)		3.64 (dd, 10, 3.6)	
4'-OCH_3_	3.80 (s)			

^a^Recorded in CD_3_OD at 600 MHz. ^b^Recorded in pyridine-*d*_5_ at 600 MHz.

**Table 2:** ^13^C–NMR data for compounds **1**–**4**.

Position	**1**^a^	**2**^b^	**3**^a^	**4**^a^

1	135.2	142.8	139.1	145.0
2	116.5	120.2	132.4	116.1
3	130.0	146.4	131.7	158.3
4	146.9	114.8	114.2	114.3
5	142.0	159.4	156.2	130.2
6	116.9	114.6	115.3	120.6
7	31.7	31.7	34.1	51.8
8	37.1	36.3	39.9	67.1
9	175.3	173.7	66.0	39.9
9-OCH_3_	52.0	51.7		
1'	135.0	143.7	149.0	143.3
2'	110.5	117.7	117.2	117.0
3'	147.4	159.2	158.5	158.1
4'	149.1	114.1	114.1	113.7
5'	116.1	130.0	130.2	130.0
6'	119.7	120.9	121.9	121.5
7'	88.8	39.5	48.0	
8'	55.7	51.7	48.1	
9'	65.1	66.7	62.5	
4'-OCH_3_	56.4			

^a^Recorded in CD_3_OD at 150 MHz. ^b^Recorded in pyridine-*d*_5_ at 150 MHz.

**Figure 2:**
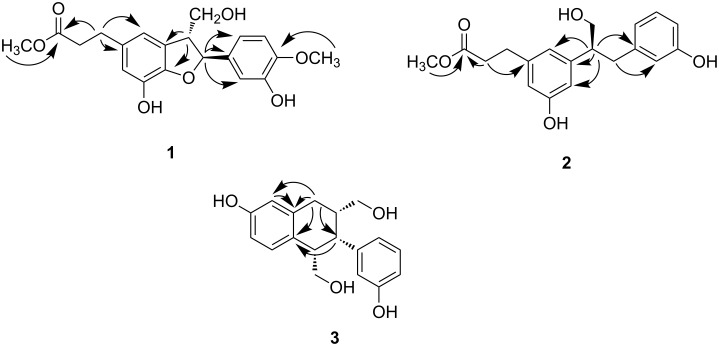
Key HMBC correlations for compounds **1**–**3**.

This compound was also presumed to have three rings in its structure based on the unsaturation degree since it contained two aromatic moieties and an ester group. ^1^H,^1^H COSY spectrum of compound **1** revealed the presence of two isolated proton spin systems of CH–CH–CH_2_ corresponding to C7′–C8′–C9′ and CH_2_–CH_2_ corresponding to C7–C8. In addition, coupling constants as well as the ^1^H,^1^H COSY data indicated the presence of a 1,3,4-trisubstituted benzene moiety at δ 6.82 (br d, *J* = 8.4 Hz, H-6′), 6.74 (d, *J* = 8.4 Hz, H-5′), and 6.95 (br s, H-2′). The extension of the spin systems and attachments of functional groups were confirmed by HMBC correlations (Figure 2).

HMBC correlations of a methoxy proton at δ_H_ 3.63 and H_2_-7 at δ_H_ 2.78 (t, *J* = 7.8 Hz) with the ester carbonyl carbon at δ_C_ 175.3 indicated the presence of a methyl propanoate. HMBC correlations of H-2–H-7 with C-1 at δ_C_ 135.2, C-2 at δ_C_ 116.5, and C-6 at δ_C_ 116.9 demonstrated that the methyl propanoate was attached to the C-1 position of one phenylpropanoid moiety in the lignan skeleton. Together with HMBC correlations, two singlet aromatic protons for H-2 and H-6 indicated that the first phenylpropanoid moiety had a 1,3,4,5-tetrasubstituted benzene ring, which was attached to the C-1 position of the methyl propanoate. H-7' at δ_H_ 5.47 (d, *J* = 6.0 Hz) of the CH–CH–CH_2_ spin system had HMBC correlations with C-1', C-2', and C-6' of the 1,3,4-trisubstituted benzene ring, indicating that the CH–CH–CH_2_ spin system was linked to C-1' of the 1,3,4-trisubstituted benzene ring. In addition, HMBC correlations of H-8' with C-3 and C-4 suggested that C-8' was linked to the C-3 position of the first phenylpropanoid moiety. HMBC correlations of H-7' with C-4 allowed linkage of the oxygenated sp^3^ methine carbon C-7' to the oxygenated sp^2^ carbon C-4 through oxygen to form a dihydrofuran ring. The presence of a dihydrofuran moiety in compound **1** was also demonstrated by chemical shifts of C-4 (δ 146.9) and C-7' (δ 88.8) together with the unsaturation requirement. Thus, compound **1** was proved to be a neolignan containing a dihydrobenzofuran skeleton. HMBC correlations of the methoxy proton at δ_H_ 3.80 with C-4' (δ 149.1) showed that the methoxy group was attached to C-4' of the 1,3,4-trisubstituted benzene ring, and a hydroxy group was presumed to be attached to C-3' based on the chemical shifts. Ultimately, the chemical structure of compound **1** was identified as shown in Figure 1 and named trogopterin A. Detailed ^1^H and ^13^C NMR data are presented in Table 1 and Table 2.

The relative stereochemistry of compound **1** was established by interpreting the NOESY data. A strong NOESY correlation between H-7' and H-9' indicated that the 1,3,4-trisubstututed benzene ring and hydroxymethylene group were on opposite faces of the molecule. The absolute configurations of C-7' and C-8' in compound **1** were established by comparing the circular dichroism (CD) data to those of previously reported neoligans containing a dihydrobenzofuran skeleton [17]. The CD of compound **1** showed positive cotton effects at 255 and 327 nm along with a negative cotton effect at 234 nm. These features were very similar to those of neolignans [17], indicating that C-7' and C-8' in compound **1** have *S* and *R* configurations, respectively. Thus, compound **1** was determined to be methyl 3-((2*S*,3*R*)-7-hydroxy-2-(3-hydroxy-4-methoxyphenyl)-3-(hydroxymethyl)-2,3-dihydrobenzofuran-5-yl)propanoate.

Compound **2** was isolated as a light brown oil. The molecular formula of compound **2** was determined to be C_19_H_22_O_5_ based on HREIMS, which gave a molecular ion peak at *m*/*z* 330.1462 (calcd 330.1467 for C_19_H_22_O_5_) indicating the presence of nine unsaturations in the structure. The ^1^H NMR spectrum of compound **2** (Table 1) was similar to that of compound **1** and contained one methoxy group (δ 3.57), a coupled sp^3^ CH_2_–CH_2_ (δ 2.93 and 2.64), and seven sp^2^ methine protons (δ 7.21–6.88). ^13^C NMR data for compound **2** demonstrated the presence of 12 sp^2^ carbons and five sp^3^ carbons together with an ester carbon, which suggested that compound **2** has a lignan skeleton similar to compound **1**. Based on the unsaturation degrees, compound **2** does not contain a ring except for two aromatic moieties. The ^1^H,^1^H COSY spectrum of compound **2** revealed the presence of two isolated proton spin systems of CH_2_–CH–CH_2_ corresponding to C7′–C8′–C9′ and CH_2_–CH_2_ corresponding to C7–C8. HMBC correlations of a methoxy proton at δ_H_ 3.57 with the ester carbon at δ_C_ 173.7 as well as H_2_-8 at δ_H_ 2.64 with C-1 at δ_C_ 142.8 indicated that a methyl propanoate group was attached to the C-1 position of one phenylpropanoid moiety of the lignan skeleton (Figure 2). HMBC correlations of the methine proton H′-8 in the CH_2_–CH–CH_2_ spin system with C-2 at δ_C_ 120.2, C-3 at δ_C_ 146.4, C-4 at δ_C_ 114.8, and C-1′ at δ_C_ 143.7 suggested that C-8′ of the second phenylpropanoid was attached to the C-3 position of the first phenylpropanoid moiety. In addition, HMBC correlation of H-7′ with C-2′ together with the coupling constant of H-2′ (1.8 Hz) supported that the second phenylpropanoid moiety has a 1,3-disubstituted benzene ring. Based on these findings, compound **2** was identified as methyl (*S*)-methyl 3-(3-hydroxy-5-(1-hydroxy-3-(3-hydroxyphenyl)propan-2-yl)phenyl)propanoate and named trogopterin B. Compound **2** was different in that it did not contain a methoxy group at the C-4′ position nor an ether linkage between C-4 and C-7′ to form a dihydrofuran ring. The absolute configuration of compound **2** was determined by comparing its optical rotation with those of (*R*)-2,3-diphenyl-1-propanol and (*S*)-2,3-diphenyl-1-propanol [18–19]. Compound **2** had a positive optical rotation value of [a]_D_^25^ +20.2, which was similar to that of (*S*)-2,3-diphenyl-1-propanol (+107°). Thus, C-8′ was determined to have *S* configuration.

Compound **3** was obtained as colorless oil. Its molecular formula was found to be C_18_H_20_O_3_ based on HREIMS (*m*/*z* 300.1360), requiring nine degrees of unsaturation. The ^1^H NMR spectrum (Table 2) indicated the presence of seven aromatic protons at δ 7.09–6.43, two oxygenated methylene protons at δ 3.66, 3.64, 3.61, and 3.39, and three sp^3^ methine protons at δ 3.84, 1.98, and 1.79. The ^13^C NMR spectrum of compound **1** indicated the presence of 12 sp^2^ carbons and six sp^3^ carbons, suggesting that compound **3** might also have a lignan skeleton similar to compounds **1** and **2**. In order to meet the requirements of unsaturation, this compound was presumed to have one more ring in addition to two aromatic moieties. The ^1^H,^1^H COSY spectrum of compound **3** revealed the presence of two isolated proton spin systems of CH_2_–CH–CH_2_ corresponding to C7–C8–C9 and CH–CH–CH_2_ corresponding to C7′–C8′–C9′. In addition, coupling constants as well as the ^1^H,^1^H COSY data [δ 6.54 (br s, H-6), 6.43 (dd, *J* = 8.4, 2.4 Hz, H-4), and 6.51 (d, *J* = 8.4 Hz, H-3)] indicated the presence of a 1,3,4-trisubstituted benzene ring. Extension of the spin systems was confirmed by HMBC correlations (Figure 2). HMBC correlations of the sp^3^ methylene protons H_2_-7 (δ 2.76) with C-1 (δ 139.1), C-2 (δ 132.4), C-6 (δ 115.3), and C-7′ (δ 48.0) indicated that the first spin system was connected to C-1 (δ 139.1) of the 1,3,4-trisubstituted benzene ring while C-8 was connected to C-7′ of the second spin system. HMBC correlations of H-7′ with C-6 (δ 115.3) together with the unsaturation requirement allowed the connection of C-8′ with C-2 to form a cyclohexene moiety. Furthermore, splitting patterns of the protons in the other aromatic moiety [6.55 (br s), 6.62 (dd, 7.8, 2.4), 7.09 (t, 7.8), and 6.64 (d, 7.8)] and downfield-shifted chemical shift of C-3′ (δ 158.5) indicated that the 3-hydroxy-1,3-disubstituted benzene ring was attached to the C-7′ carbon. In this way, the planar structure of compound **3** was elucidated as shown in Figure 1.

**Figure 3:**
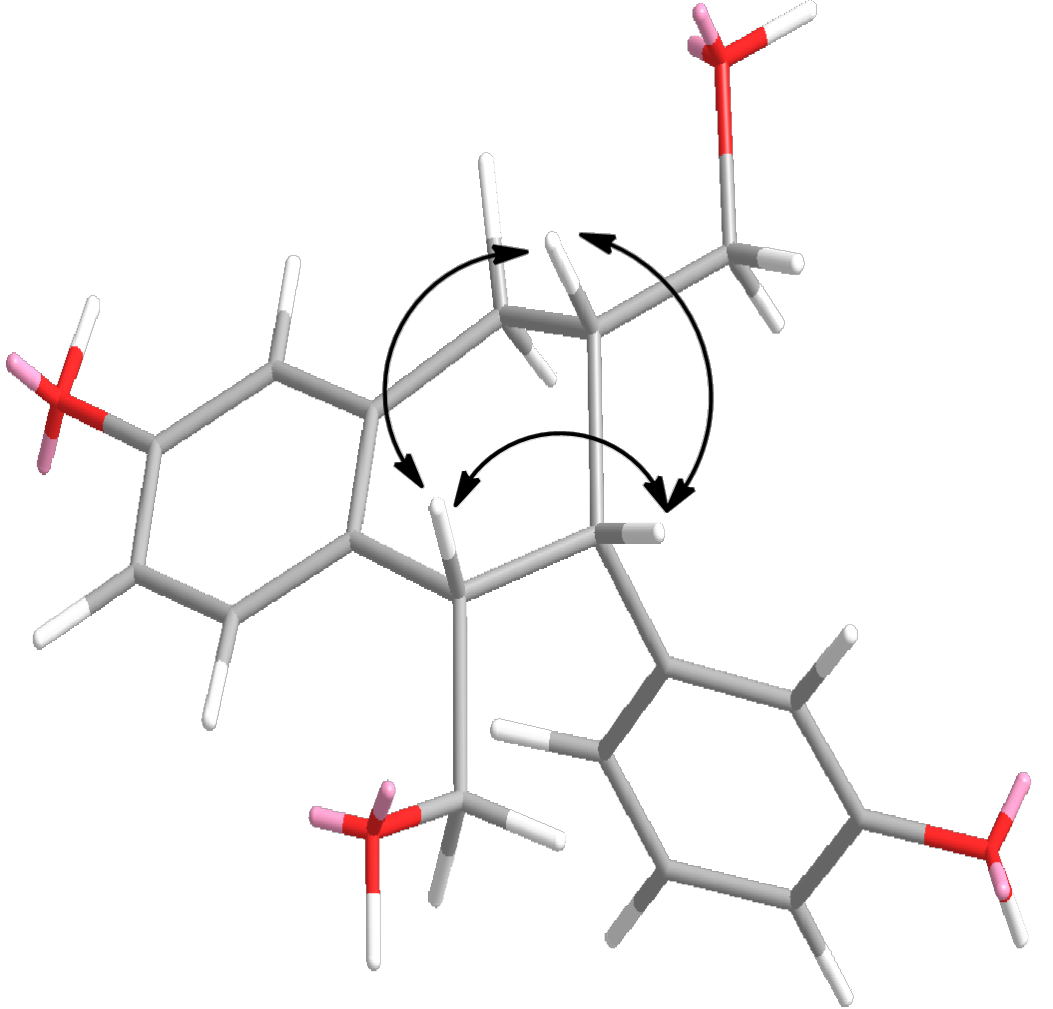
Key NOESY correlations (^1^H↔^1^H) for trogopterin C (compound **3**) identified in the ChemDraw 3D MM2-minimized model.

The relative stereochemistry of compound **3** was deduced based on NOESY data and comparison with interproton distances calculated by MM2 with ChemDraw. A strong NOESY correlation between H-7′ and H-8′ indicated that the phenyl group at C-7′ and the hydroxymethylene group at C-8′ are on the same face of the molecule (Figure 3). Additionally, a strong NOESY correlation between H-8 and H-8′ indicated that the hydroxymethylene group at C-8 and the hydroxymethylene group at C-8′ are on the same face of the molecule (Figure 3). A structure model of this compound was created using MM2 with ChemDraw 3.0 (Figure 3).

The resulting calculated interproton distances were in close agreement with the NOESY data (Table 3).

**Table 3:** Interproton distances (Å) for trogopterin C (compound **3**) in the MM2-minimized model.

Proton	To proton	Interproton distance

H-8	H-7'	2.426
H-8	H-8'	2.467
H-7'	H-8'	2.314
H-7'	H-9'a	4.007
H-7'	H-9'b	4.309
H-7'	H-2'	2.543

A careful modeling study considering all the possible conformers of compound **3** exhibited that interproton distances between H-7, H-8, and H-8′ are within 3 Å when two hydroxymethylene groups and a hydroxyphenyl group in compound **3** are on the same face of the molecule. Thus, the structure of compound **3** was determined to be ((1*RS*,2*RS*,3*RS*)-6-hydroxy-2-(3-hydroxyphenyl)-1,2,3,4-tetrahydronaphthalene-1,3-diyl)dimethanol and named trogopterin C.

Compound **4** was isolated as light brown oil. The ^1^H and ^13^C NMR spectra contained signals similar to those of compound **2** except for those corresponding to the methyl propanoate moiety in compound **2**. Based on the ^1^H, ^13^C, HMQC, and HMBC data, compound **4** had an aromatic proton instead of the methyl propanoate found in compound **2**. Thus, compound **4** was determined to be ((*R*)-3,3'-(3-hydroxypropane-1,2-diyl)diphenol as previously reported [13]. Even though this compound has been described in the literature, the absolute stereochemistry was not elucidated. Similar to compound **2**, the optical rotation of compound **4** was compared to those of (*R*)-2,3-diphenyl-1-propanol and (*S*)-2,3-diphenyl-1-propanol [18–19]. Compound **4** had a negative optical rotation of [a]_D_^25^ −15.4 similar to (*R*)-2,3-diphenyl-1-propanol [19]. Thus, the structure of **4** was identified as (*R*)-3,3'-(3-hydroxypropane-1,2-diyl)diphenol.

The in vitro cytotoxic activities of compounds **1**–**4** against HL-60 (human leukemia), HeLa (human cervical carcinoma), and MCF-7 (human breast cancer) cells were evaluated using an MTT assay. As shown in Table 4, compounds **1**–**3** exerted moderate cytotoxic effects against HL-60 cells with IC_50_ values of 34.77–45.68 μM using adriamycin as a positive control (IC_50_ = 0.18 μM). Additionally, compound **1** showed very weak cytotoxic activity against MCF-7 cells with an IC_50_ of 94.69 μM. None of the compounds affected the HeLa cells.

**Table 4:** Cytotoxic effects of compounds **1**–**4** against HL-60, HeLa, and MCF-7 cells.

Compound	IC_50_ (μM)		

	HL- 60	HeLa	MCF-7

1	45.68 ± 3.25	>100	>100
2	34.77 ± 3.12	>100	>100
3	42.18 ± 4.35	>100	94.69 ± 10.25
4	67.94 ± 4.12	>100	>100
Adriamycin	0.18 ± 0.11	2.30 ± 0.57	4.25 ± 1.02

## Conclusion

In summary, two novel neoligans (trogopterins A and B) and a new phenolic compound (trogopterin C) were isolated from the crude methanol extract of *Trogopterus* feces for the first time. The absolute configurations of compounds **1**, **2**, and **4** were determined by comparing CD spectra and optical rotations. The levels of cytotoxicity against three tumor cell lines (HL-60, HeLa, and MCF-7) were evaluated. Compounds **1**–**3** had moderate cytotoxic effects against HL-60 cells even though the activities were not significant. Thus, *Trogopterus* feces could be a potential source of lignans with cytotoxic activity.

## Experimental

### General Methods

Optical rotations were measured with a Jasco P1000 digital polarimeter. UV spectra were recorded by a Hewlett Packard 8453 UV–vis spectrometer. CD spectra were obtained with a Jasco J-715 circular dichroism spectrophotometer. 1D and 2D NMR were performed on a VNS 600 MHz spectrometer operating at 600 MHz for protons and 150 MHz for carbon. Chemical shifts are expressed in ppm and referenced relative to the residual solvent signals. Mass spectra were recorded with a Micromass LCT mass spectrometer, and the lock mass calibration was applied to accurately measure masses. Semi-preparative HPLC was performed with an Agilent system consisting of a vacuum degasser, quaternary pump, diode array detector (DAD), and Luna 5u C_18_ (2) 100 Å column (250 × 10.00 mm; Phenomenex). Column chromatography (CC) was carried out on either silica gel (Merck KGaA, 70–230 mesh) or Sephadex^TM^ LH-20 (GE Healthcare Sweden). TLC was performed with precoated silica gel 60 F254 plates (Merck KGaA) and spots were visualized under UV light (254 and 365 nm) or by spraying with 20% H_2_SO_4_ and heating.

### Material

Dried *T. xanthipes* feces were provided by the oriental hospital of Dongguk University (Seoul, South Korea). A voucher specimen (No. YU-BT-2013-03) was deposited in the Natural Products Chemistry Laboratory of the School of Biotechnology, Yeungnam University (Gyeongsan, South Korea).

### Extraction and isolation

The air-dried Trogopterus feces (1 kg) were subjected to extraction three times (3 h per cycle) with refluxing methanol. The solvent was evaporated under reduced pressure to recover the methanolic extracts (98 g) that were partitioned successively between H_2_O and CHCl_3_ and EtOAc. The EtOAc extract (6.5 g) was subjected to silica gel CC (5 × 60 cm column) using a gradient of methylene chloride (MC) and acetonitrile (ACN) as eluents to acquire 27 fractions (Fr. 1–27). Fr. 16 (74 mg) was further separated with Sephadex^TM^ LH-20 and methanol to recover eight subfractions (SFr. 16-1–16-8). SFr. 16-4 was further subjected to semi-preparative reverse-phase HPLC (Luna 5u Phenomenex column; 250 × 10.00 mm; flow rate, 2 mL/min; 20–40% ACN in H_2_O for 20 min, 40–50% for 15 min, 50–100% for 10 min; UV detection at 254 nm) to collect compound **1** (3.5 mg, *t*_R_ = 24.7 min). Fr. 17 (27.2 mg) underwent semi-preparative reversed-phase HPLC using the same method as that performed to recover compound **1**; this yielded compounds **2** (15.0 mg, *t*_R_ = 22.1 min) and **4** (1.7 mg, *t*_R_ = 19.3 min). Fr. 23 (184.7 mg) was also separated by silica gel CC to collect 15 subfractions (SFr. 23-1–23-15). Compound **3** (3.2 mg, *t*_R_ = 15.1 min) was obtained from SFr. 23-10 (12 mg) by semi-preparative reversed-phase HPLC (Luna 5u Phenomenex column; 250 × 10.00 mm; flow rate, 2 mL/min; 20–50% ACN in H_2_O for 20 min, 50–65% for 20 min, 65–100% for 10 min; UV detection at 254 nm). Fr. 3 (58 mg) was subjected to semi-preparative reversed-phase HPLC (Luna 5u C18 column; 250 × 10.00 mm; flow rate, 2 mL/min; 15% ACN in H_2_O for 20 min, 15–65% for 15 min, 65–100% for 5 min; UV detection at 254 nm) to afford compounds **5** (4.5 mg, *t*_R_ = 7.5 min) and **6** (2.3 mg, *t*_R_ = 12.3 min). The CHCl_3_-soluble layer (4.5 g) was subjected to silica gel CC (5 × 60 cm column) using a gradient of *n*-hexane and ethyl acetate (EtOAc) as eluents to recover 12 fractions (HF. 1–12). Fraction 3 (86 mg) was further separated by silica gel CC (5 × 60 cm column) using a gradient of *n*-hexane, EtOAc, and methanol to collect compound **7**. In addition, HF. 7 was further subjected to semi-preparative reverse-phase HPLC (Luna 5u Phenomenex column; 250 × 10.00 mm; flow rate, 2 mL/min; 40–80% ACN in H_2_O for 35 min, 80–100% for 10 min; UV detection at 220 nm) to recover compound **7**.

### Characterization

Trogopterin A (**1**): light brown oil; [α]_D_^25^ +33.62 (*c* 1.0 × 10^−3^ g/mL, EtOH); CD (MeOH) 234 (∆ε −2.53), 255 (∆ε +0.92), 327 (∆ε +2.12); ^1^H and ^13^C NMR (600 and 150 MHz,CD_3_OD), see Table 1. HMBC correlations (CD_3_OD, H-#→C-#) H-2→C-4, C-6, C-7, and C-8'; H-6→C-2, C-4, C-5, and C-7; H-7→C-1, C-2, C-8, and C-9; H-8→C-1, C-7, and C-9; H-2'→C-1', C-3', C-4', C-6', and C-7'; H-5'→C-1', C-3', and C-4'; H-6'→C-2', C-4', and C-7'; H-7'→C-3, C-4, C-1', C-2', C-6', C-8', and C-9'; H-8'→C-3, C-4, C-1', C-7', and C-9'; H-9'a→C-3, C-7', and C-8'; H-9'b→C-3, C-7', and C-8'; 9-OC*H*_3_→C-9; 4'-OC*H*_3_→C-4'; ^1^H,^1^H COSY correlations (CD_3_OD, H-#↔H-#) H-7↔H-8, H-5'↔H-6', H-7'↔H-8', H-8'↔H-9'a and H-9'b, H-9'a↔H-8' and H-9'b; NOESY correlations (CD_3_OD, H-#↔H-#) H-2↔H-8' and H-9', H-6'↔H-5', H-7', and H-8'; H-7'↔H-2', H-8', and H-9'; H-8'↔H-2' and H-9'; HREIMS (*m*/*z*): calcd for C_20_H_22_O_7_, 374.1365; found, 374.1364.

Trogopterin B (**2**): light brown oil; [α]_D_^25^ +20.2 (*c* 1.0 × 10^−3^ g/mL, EtOH); ^1^H and ^13^C NMR (600 and 150 MHz, pyridine-*d*_5_) spectroscopic analysis, see Table 1. HMBC correlations (pyridine-*d*_5_, H-#→C-#) H-2→C-4, C-6, and C-7; H-4→C-2, C-6, and C-8'; H-6→C-2, C-4, and C-7; H-7→C-1, C-2, C-6, and C-9; H-8→C-1 and C-9, H-2'→C-4' and C-6', H-4'→C-2' and C-6', H-5'→C-1', C-3', C-4', and C-6'; H-6'→C-2' and C-4', H-7'a→C-5, C-1', C-2', and C-6'; H-7'b→C-5, C-1', C-2', and C-6'; H-8'→C-2, C-3, C-4, and C-1'; H-9'→C-3, C-7', and C-8'; 9-OCH_3_→C-9; ^1^H,^1^H COSY correlations (pyridine, H-#↔H-#) H-7↔H-8, H-4'↔H-5', H-5'↔H-6', H-7'a↔H-7'b and H-8', H-7'b↔H-8', H-8'↔H-9'; HREIMS (*m*/*z*): calcd for C_19_H_22_O_5_, 330.1467; found, 330.1469.

Trogopterin C (**3**): light brown oil; [α]_D_^25^ −14.8 (*c* 1.0 × 10^−3^ g/mL, EtOH); ^1^H and ^13^C NMR (600 and 150 MHz, CD_3_OD) spectroscopic analysis, see Table 2. HMBC correlations (CD_3_OD, H-#→C-#): H-1→C-5 and C-9, H-3→C-1 and C-5, H-4→C-2, C-7, and C-10; H-6→C-12, H-7→C-4, C-5, C-6, C-10, C-12, C-1', C-2', and C-6'; H-8→C-6 and C-7, H-9→C-7, C-8, and C-11; H-11a→C-7, C-8, and C-9; H-11b→C-7, C-8, and C-9; H-12a→C-6, C-7, and C-8; H-12b→C-6, C-7, and C-8; H-2'→C-6, C-3', C-4', and C-6'; H-4'→C-2' and C-6', H-5'→C-1' and C-3', H-6'→C-6, C-2', and C-4'; ^1^H-^1^H COSY correlations (CD_3_OD, H-#↔H-#) H-6↔H-7, H-12a, and H-12b; H-8↔H-9, H-11a, and H-11b; H-4'↔H-5', H-5'↔H-6'; NOESY correlations (CD_3_OD, H-#↔H-#): H-7↔H-8, H-8', and H-9'a; H-8↔ H-7', H-8', H-9'a, and H-9'b; H-5'↔H-4' and H-6'; H-6'↔H-5', H-7', and H-8'; H-7'↔H-8' and H-9'a; H-8'↔H-9'a and H-9'b; H-9'a↔H-9'b; HREIMS (*m*/*z*): calcd for C_18_H_20_O_3_, 300.1362; found, 300.1360.

3-(1-hydroxy-3-(3-hydroxyphenyl)propan-2-yl)phenol (**4**): light brown oil; [α]_D_^25^ −15.4 (*c* 1.0 × 10^−3^ g/mL, EtOH); ^1^H and ^13^C NMR (600 and 150 MHz, pyridine-*d*_5_) spectroscopic analysis, see Table 2. HMBC correlations (CD_3_OD, H-#→C-#) H-2→C-3, C-6, and C-7; H-4→C-2, C-3, and C-6; H-5→C-1 and C-3; H-6→C-2, C-4, and C-7; H-7→C-1, C-2, C-6, C-8, C-9, and C-1'; H-8→C-1, C-7, and C-9; H-9a→C-1, C-7, C-8, C-1', C-2', and C-6'; H-9b→C-1, C-7, C-8, C-1', C-2', and C-6'; H-2'→C-9, C-3', C-4', and C-6'; H-4'→C-2', and C-6'; H-5'→C-1' and C-3', H-6'→C-9, C-2', and C-4'; HREIMS (*m*/*z*): calcd for C_15_H_16_O_3_, 244.1099; found, 244.1105.

### Assessment of cytotoxicity

Different types of cancer cells (HL-60, HeLa, and MCF-7) were maintained in RPMI 1640 medium supplemented with L-glutamine, 10% fetal bovine serum (FBS), and 2% penicillin–streptomycin. The cells were cultured at 37 °C in a 5% CO_2_ incubator. Cytotoxic activity was measured using a modified MTT assay [20]. Viable cells were seeded with the growth medium (100 µL) in 96-well microtiter plates (1 × 10^4^ cells per well) and incubated at 37 °C in a 5% CO_2_ incubator. The test sample was dissolved in DMSO for the final sample concentrations to be adjusted from 5.0 to 150 µM by diluting with the growth medium. Each sample was prepared in triplicate. The final DMSO concentration was adjusted to be below 0.1%. After standing for 24 h, 10 µL of the test sample was added to each well. The same volume of DMSO alone was added to the control wells. The medium was removed after 48 h of incubation with the test samples, and 10 µL of MTT were then added to the each well (final concentration, 5 mg/mL). After an additional 4 h of incubation, the resulting formazan crystals were dissolved in 150 mL of DMSO and the optical density (O.D.) was measured at 570 nm. The IC_50_ value was defined as the concentration of sample that reduced absorbance by 50% relative to the vehicle-treated control. Adriamycin was used as a positive control.

## Supporting Information

File 1 NMR and MS spectra of compounds.

## References

[1] Lee H, Kim Y, Choi I, Min B S, Shim S H (2010). Bioorg Med Chem Lett.

[2] Shim S H, Baltrusaitis J, Gloer J B, Wicklow D T (2011). J Nat Prod.

[3] Tang X G, Huang W Q J (2008). Emerg Tradi Chin Med.

[4] Yang N-Y, Tao W-W, Duan J-A (2009). J Asian Nat Prod Res.

[5] Yang D M, Su S W, Li X, Zhu R (1987). Acta Pharm Sin.

[6] Numata A, Yang P, Takahashi C, Fujiki R, Nabae M, Fujita E (1989). Chem Pharm Bull.

[7] Numata A, Takahashi C, Miyamoto T, Yoneda M, Yang P M (1990). Chem Pharm Bull.

[8] Yang N-Y, Tao W-W, Zhu M, Duan J-A, Jiang J-G (2010). Fitoterapia.

[9] Zhao J, Zhu H-J, Zhou X-J, Yang T-H, Wang Y-Y, Su J, Li Y, Cheng Y-X (2010). J Nat Prod.

[10] Jiao Y, Li D, Liu X Q, Shi (2009). C Zhongyaocai.

[11] Yang N-Y, Tao W-W, Duan J-A (2010). Nat Prod Res.

[12] Baek S Y, Shim S H (2012). Planta Med.

[13] Mei W, Liu Z, Li X, Dai H (2010). Redai Yaredai Zhiwu Xuebao.

[14] Yang H O, Suh D-Y, Han B H (1995). Planta Med.

[15] Guerrero I C, Andrés L S, León L G, Machin R P, Padrón J M, Luis J G, Delgadillo J (2006). J Nat Prod.

[16] Chamy M C, Piovano M, Garbarino J A, Miranda C, Gambaro V (1990). Phytochemistry.

[17] Yuen M S M, Xue F, Mak T C W, Wong H N C (1998). Tetrahedron.

[18] Buchan R, Watson M B (1968). J Chem Soc C.

[19] Podestá J C, Chopa A B, Koll L C, Mandolesi S D (1992). J Organomet Chem.

[20] Kim D C, Kim J A, Min B S, Jang T-S, Na M, Lee S H (2010). Helv Chim Acta.

