# Supplementary material for: Trogopterins A–C: Three new neolignans from feces of Trogopterus xanthipes
Source: Beilstein J Org Chem. 2014 Dec 11;10:2955–62. doi: 10.3762/bjoc.10.313 (PMC4273270; doi:10.3762/bjoc.10.313)
Supplement: File 1 — NMR and MS spectra of compounds. [file Beilstein_J_Org_Chem-10-2955-s001.pdf]

Supporting Information

for

Trogopterins A–C: Three new neolignans from feces of *Trogopterus xanthipes*

Soyoon Baek[§,1], Xuikui Xia[§,1,2], Byung Sun Min[3], Chanil Park[*,¶,4] and Sang Hee Shim[*,¶,1]

Address: [1]School of Biotechnology, Yeungnam University, Daedong, Gyeongsan, Gyeongbuk 712-749, South Korea, [2]Key Laboratory for Applied Microbiology of Shandong Province, Biotechnology Center of Shandong Academy of Sciences, Jinan 250014, P. R. China, [3]College of Pharmacy, Catholic University of Daegu, 13-13 Hayang-ro, Gyeongsan, Gyeongbuk 712-702, South Korea and [4]Department of Marine Biology & Aquaculture, Gyeongsang National University, Tongyeong, Gyeongnam 650-160, South Korea

Email: Sang Hee Shim[*]-shshim29@ynu.ac.kr; Chanil Park[*]-vinus96@hanmail.net

[*]Corresponding author

[§]These authors equally contributed to this work.

[¶]Tel. Sang Hee Shim-+82 53 810 3028; Chanil Park-+82 55 772 9153

Contents

Figure S1: ^{1}H NMR spectrum of compound **1** (DMSO-d_6, 600 MHz)

Figure S2: ^{13}C NMR spectrum of compound **1** (DMSO-d_6, 150 MHz)

Figure S3: $^{1}H,^{1}H$ COSY spectrum of **1** (DMSO-d_6, 600 MHz)

Figure S4: HMQC spectrum of compound **1** (DMSO-d_6, 600 MHz)

Figure S5: HMBC spectrum of compound **1** (DMSO-d_6, 600 MHz)

Figure S6: NOESY spectrum of compound **1** (DMSO-d_6, 600 MHz)

Figure S7: HREIMS of compound **1**

Figure S8: ^{1}H NMR spectrum of compound **2** (pyridine-d_5, 600 MHz)

Figure S9: ^{13}C NMR spectrum of compound **2** (pyridine-d_5, 150 MHz)

Figure S10: $^{1}H,^{1}H$ COSY spectrum of **2** (pyridine-d_5 , 600 MHz)

Figure S11: HMQC spectrum of compound **2** (pyridine-d_5 , 600 MHz)

Figure S12: HMBC spectrum of compound **2** (pyridine-d_5 , 600 MHz)

Figure S13: HREIMS of compound **2**

Figure S14: ^{1}H NMR spectrum of compound **3** (CD_3OD, 600 MHz)

Figure S15: ^{13}C NMR spectrum of compound **3** (CD_3OD, 150 MHz)

Figure S16: $^{1}H,^{1}H$ COSY spectrum of **3** (CD_3OD, 600 MHz)

Figure S17: HMQC spectrum of compound **3** (CD_3OD, 600 MHz)

Figure S18: HMBC spectrum of compound **3** (CD_3OD, 600 MHz)

Figure S19: NOESY spectrum of compound **3** (CD_3OD, 600 MHz)

Figure S20: HREIMS of compound **3**

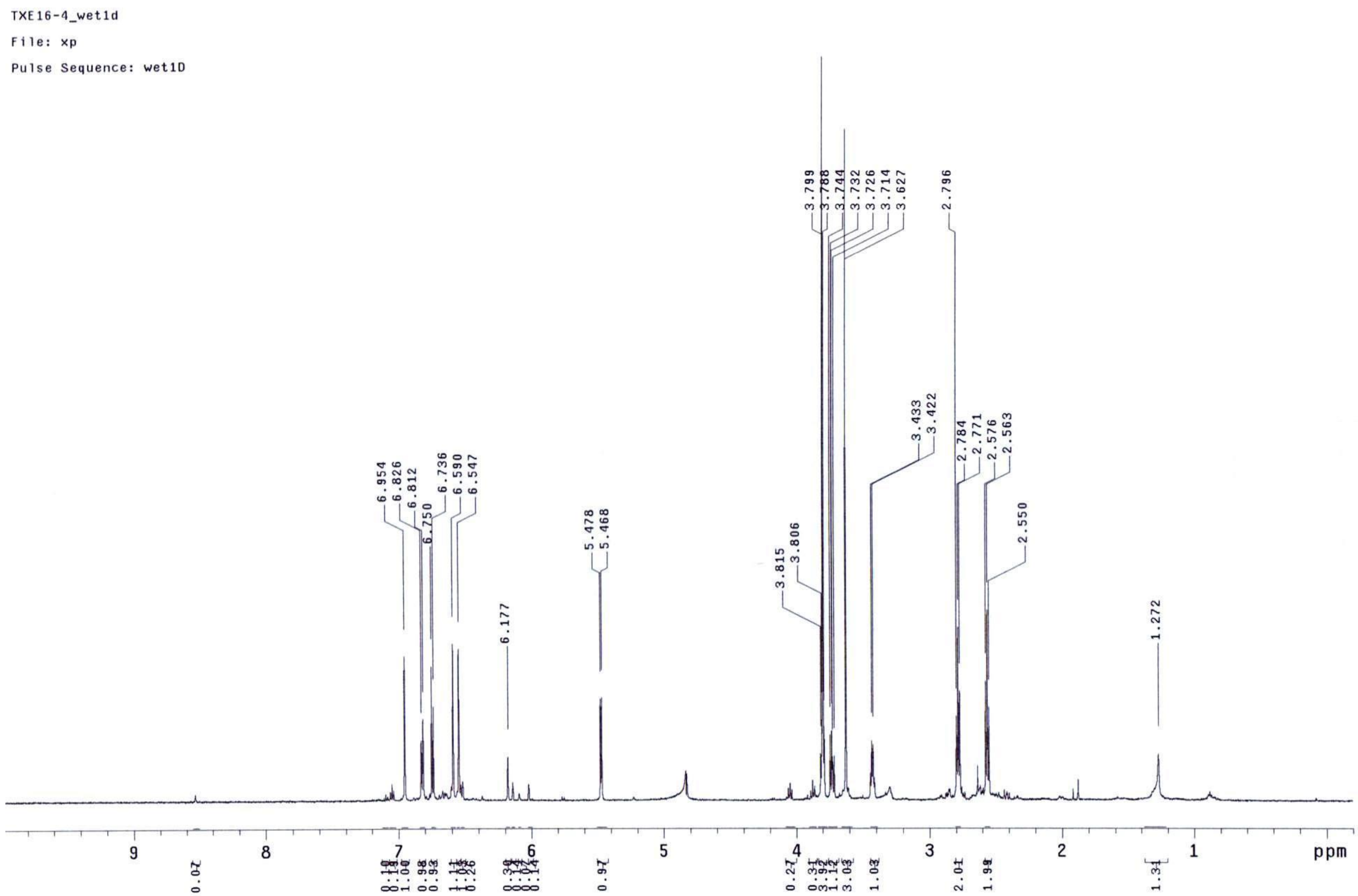

Figure S1: ¹H NMR spectrum of compound **1** (DMSO-d_6, 300 MHz).

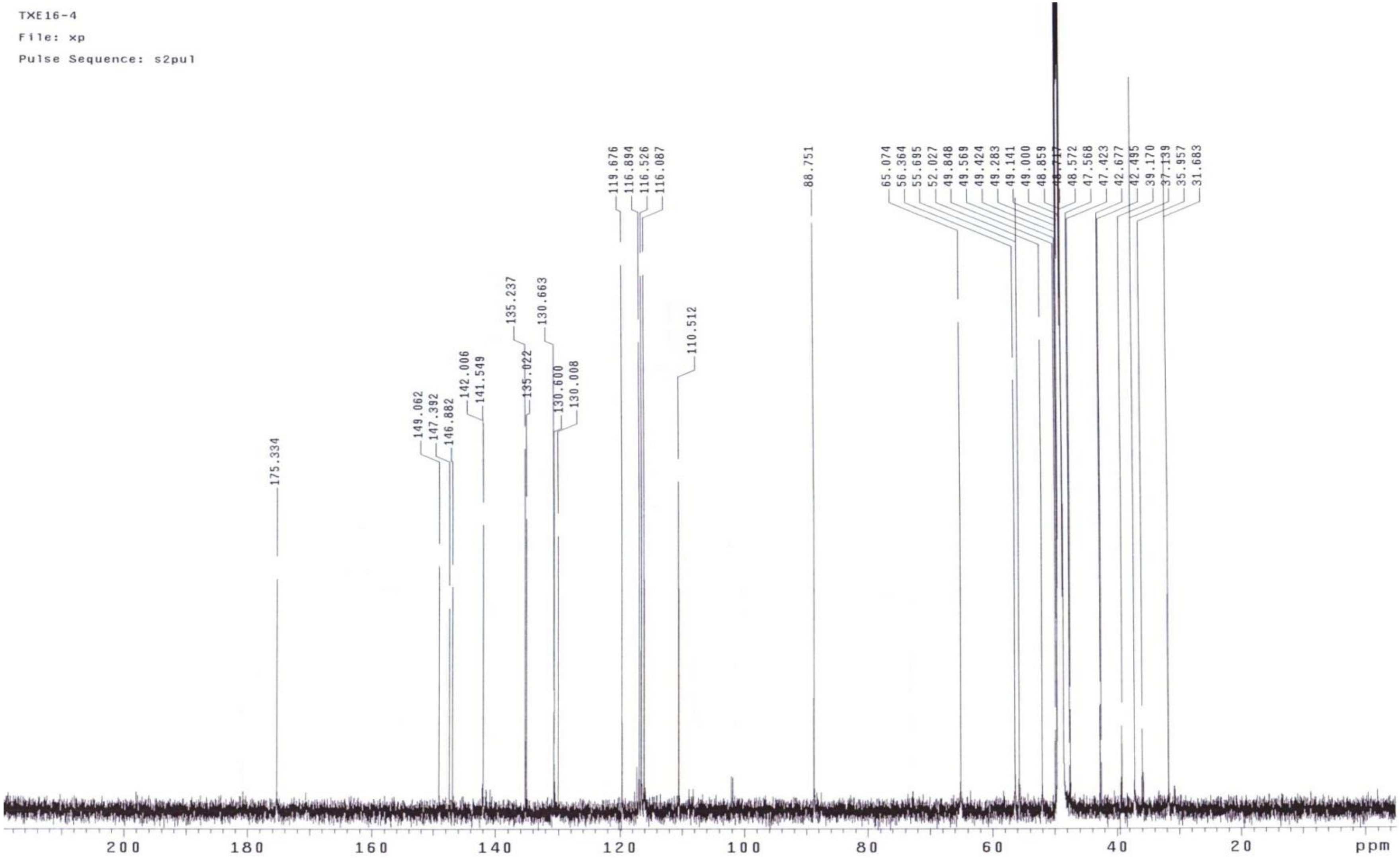

Figure S2: ^{13}C NMR spectrum of compound **1** (DMSO-d_6, 75 MHz).

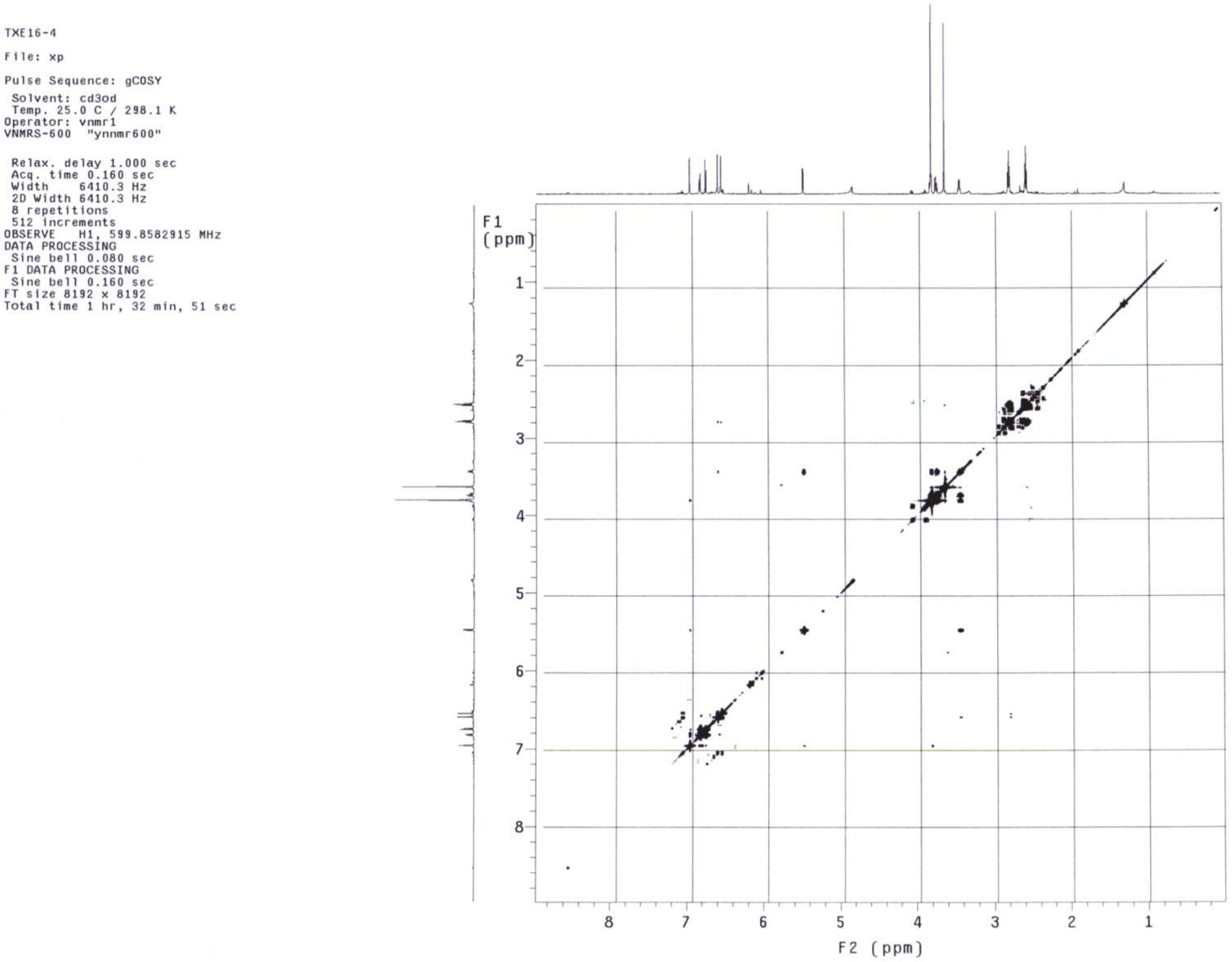

Figure S3: $^{1}H,^{1}H$ COSY spectrum of compound **1** (DMSO-d_6, 600 MHz).

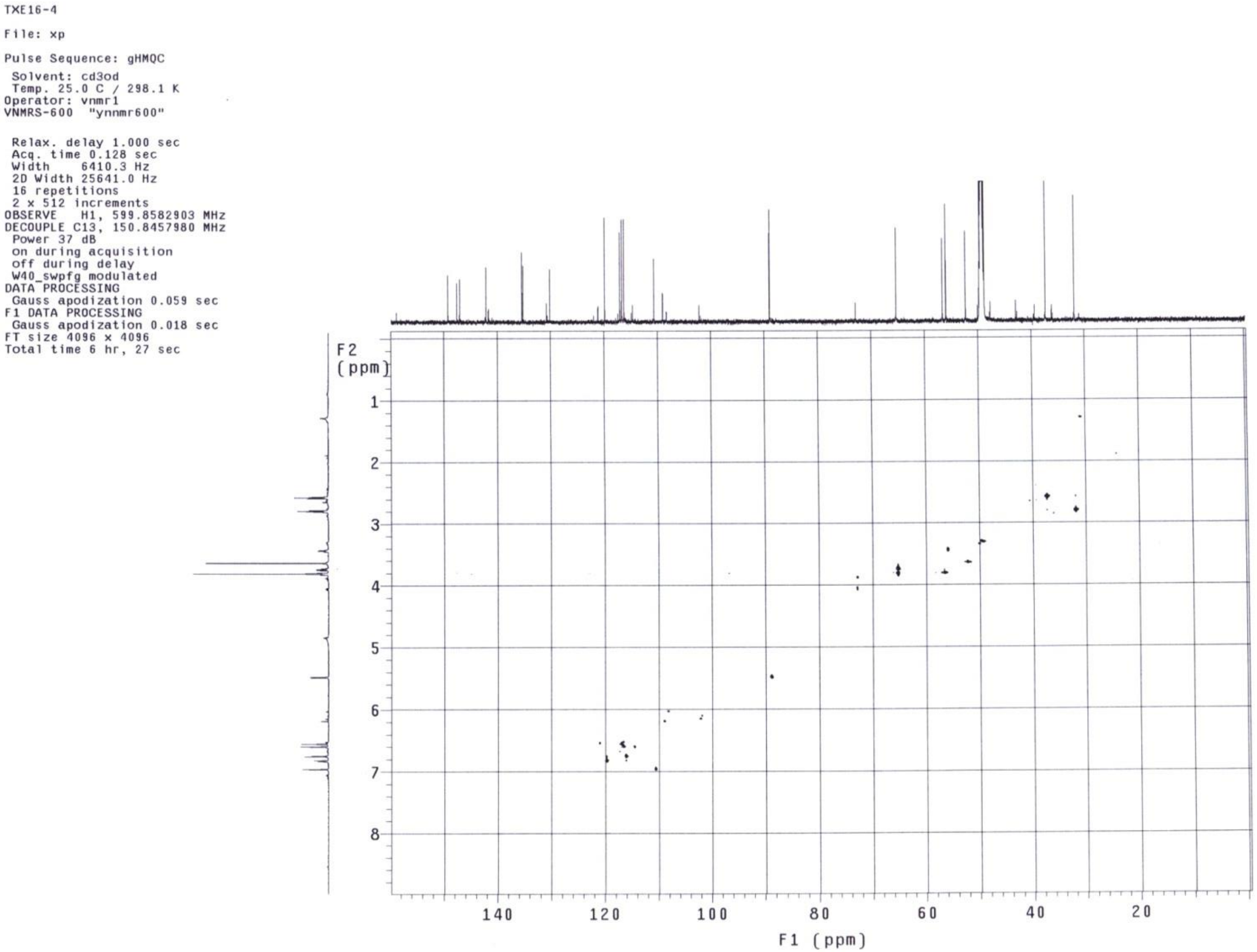

Figure S4: HMQC spectrum of compound **1** (DMSO-d_6, 600 MHz).

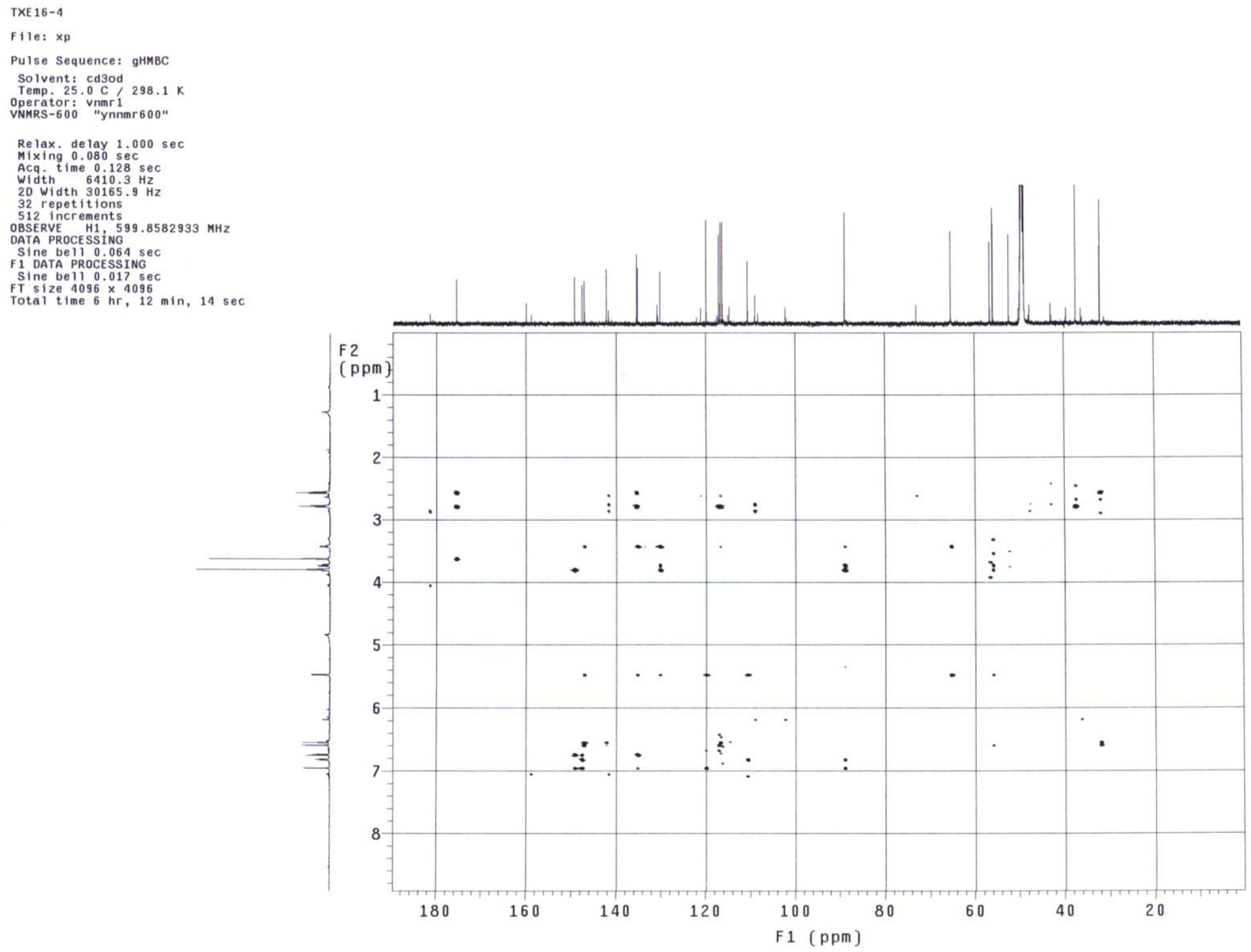

Figure S5: HMBC spectrum of compound **1** (DMSO-d_6, 600 MHz).

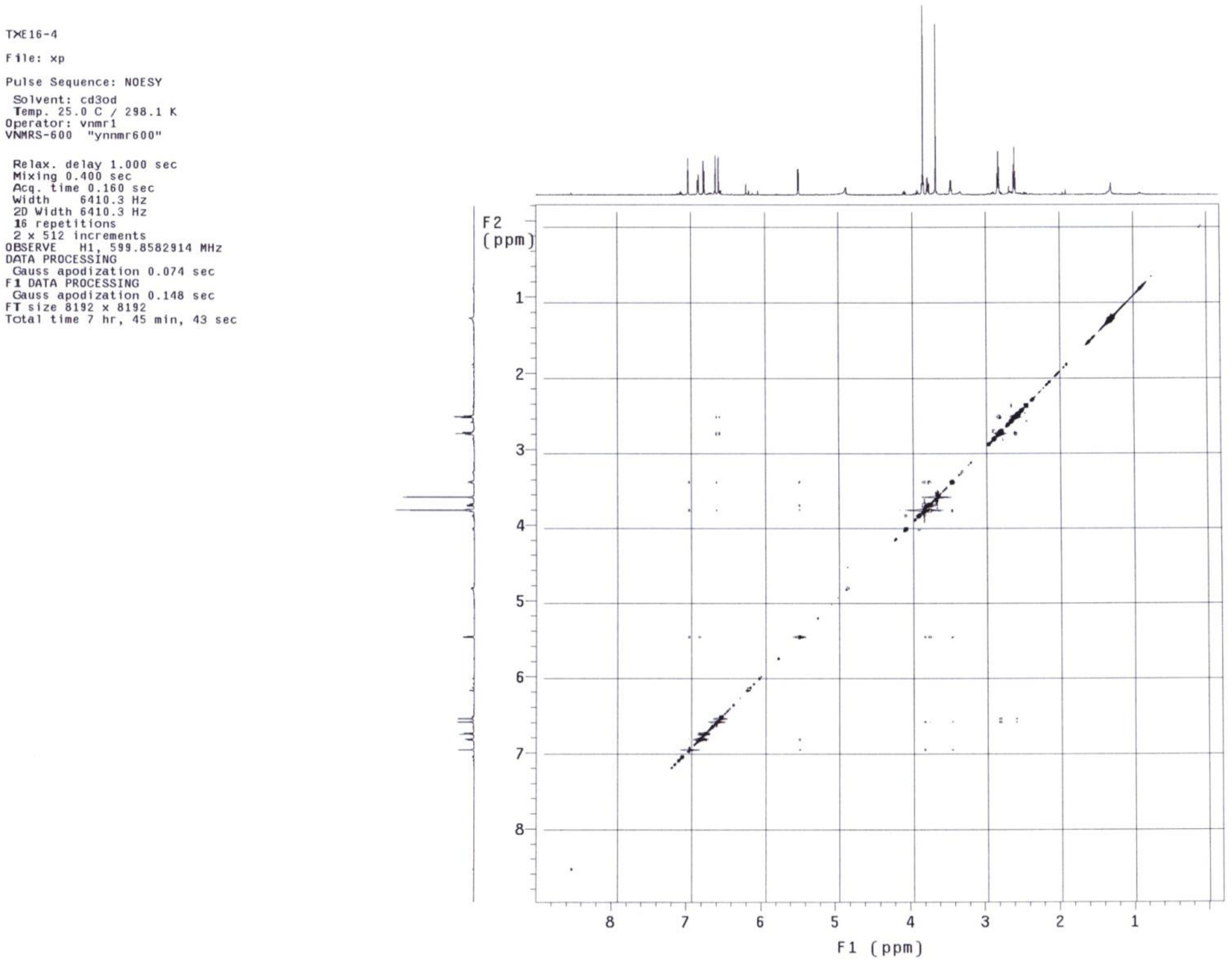

Figure S6: NOESY spectrum of compound **1** (DMSO-d_6, 600 MHz).

File: 10221
Sample: TXE-1

Date Run: 12-12-2013
Ionization mode: EI+

Time Run: 11:21:59

Instrument: JEOL JMS600
Inlet: Direct Probe

R.T.: 1:45.6

Scan: 139-163
Base: m/z 344; 6.1%FS

TIC: 1084381

Selected Isotopes : $C_{0\text{-}50}H_{0\text{-}100}O_{0\text{-}10}$

Error Limit : 20 ppm

Measured Mass	*% Base*	*Formula*	*Calculated Mass*	*Error*
**374.1364**	**39.8%**	$C_{27}H_{18}O_2$	**374.1307**	**-15.0**
		$C_{20}H_{22}O_7$	**374.1365**	**0.4**

Figure S7: HREIMS of compound **1.**

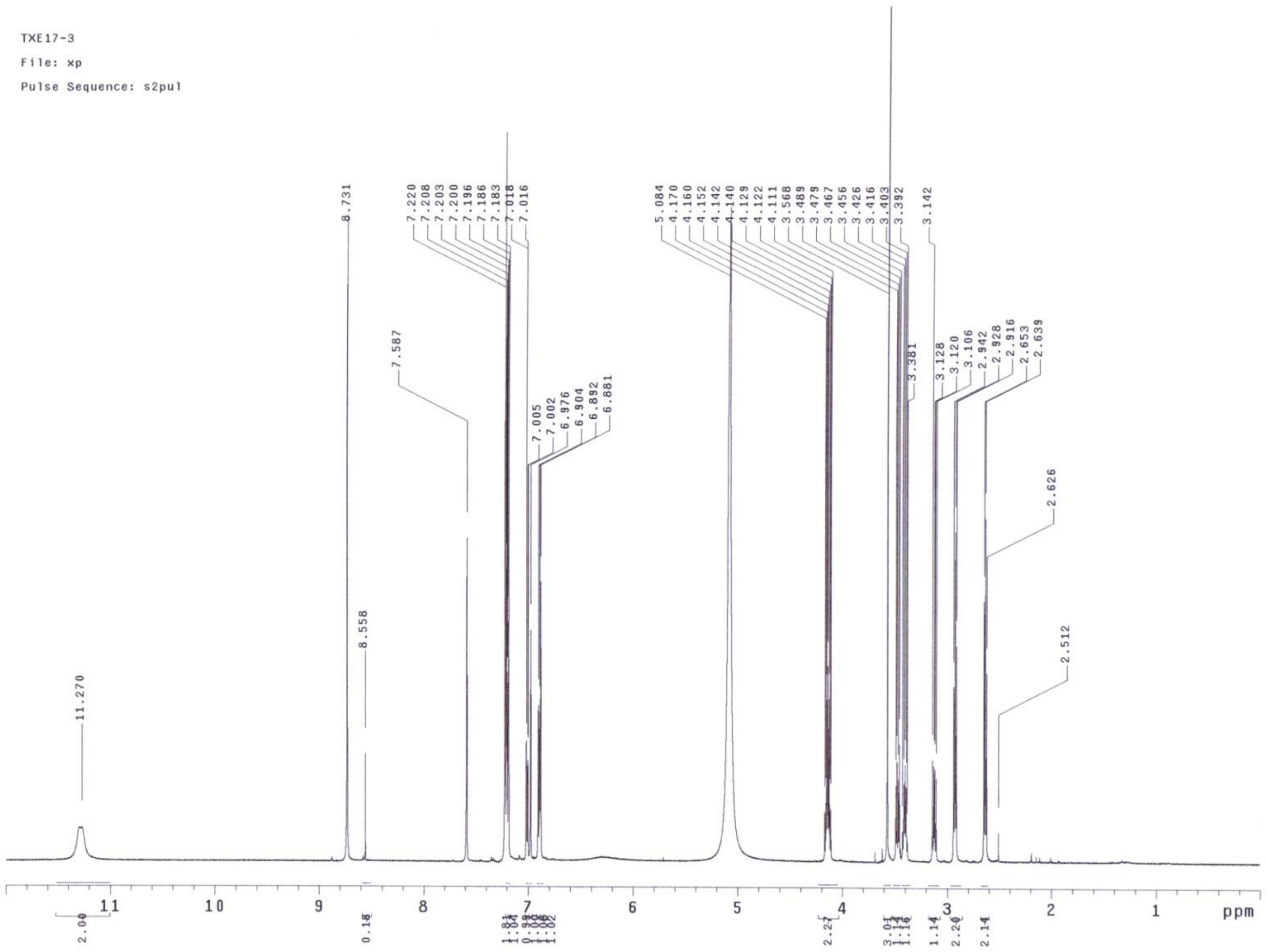

Figure S8: ^{1}H NMR spectrum (pyridine-d_5, 600 MHz) of compound **2.**

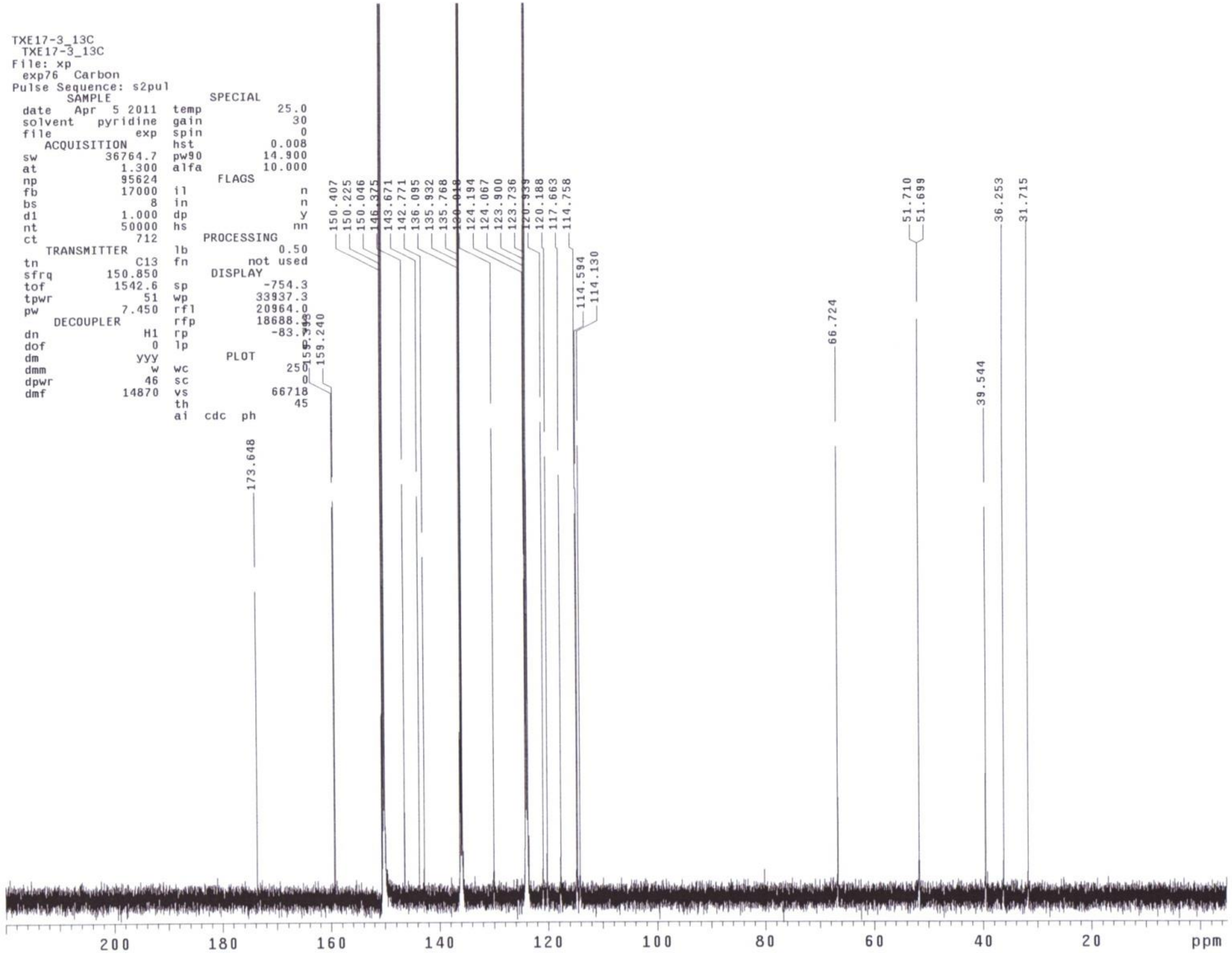

Figure S9: ^{13}C NMR spectrum (pyridine-d_5, 150 MHz) of compound **2.**

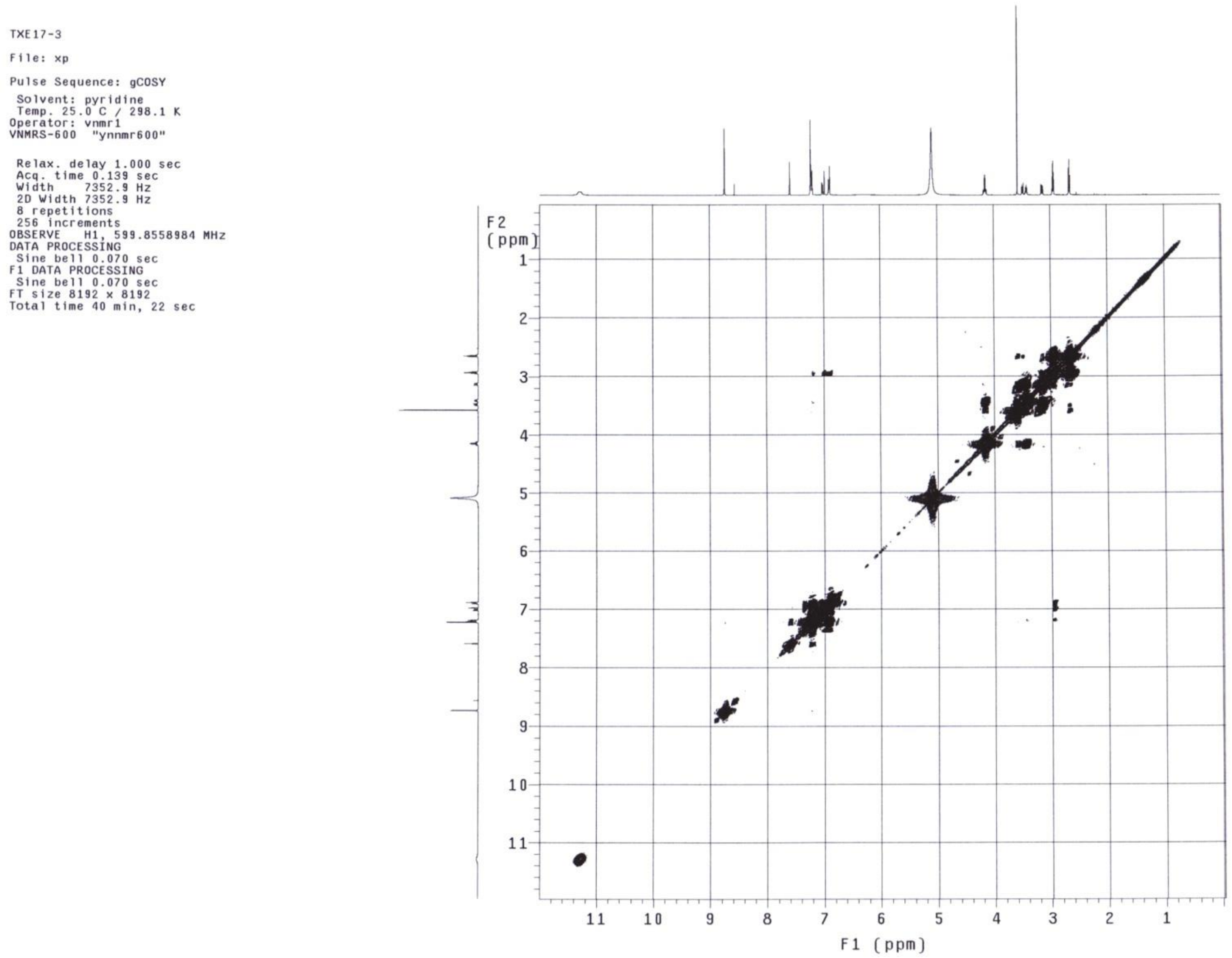

Figure S10: $^{1}H,^{1}H$ COSY spectrum (pyridine-d_5, 600 MHz) of compound **2.**

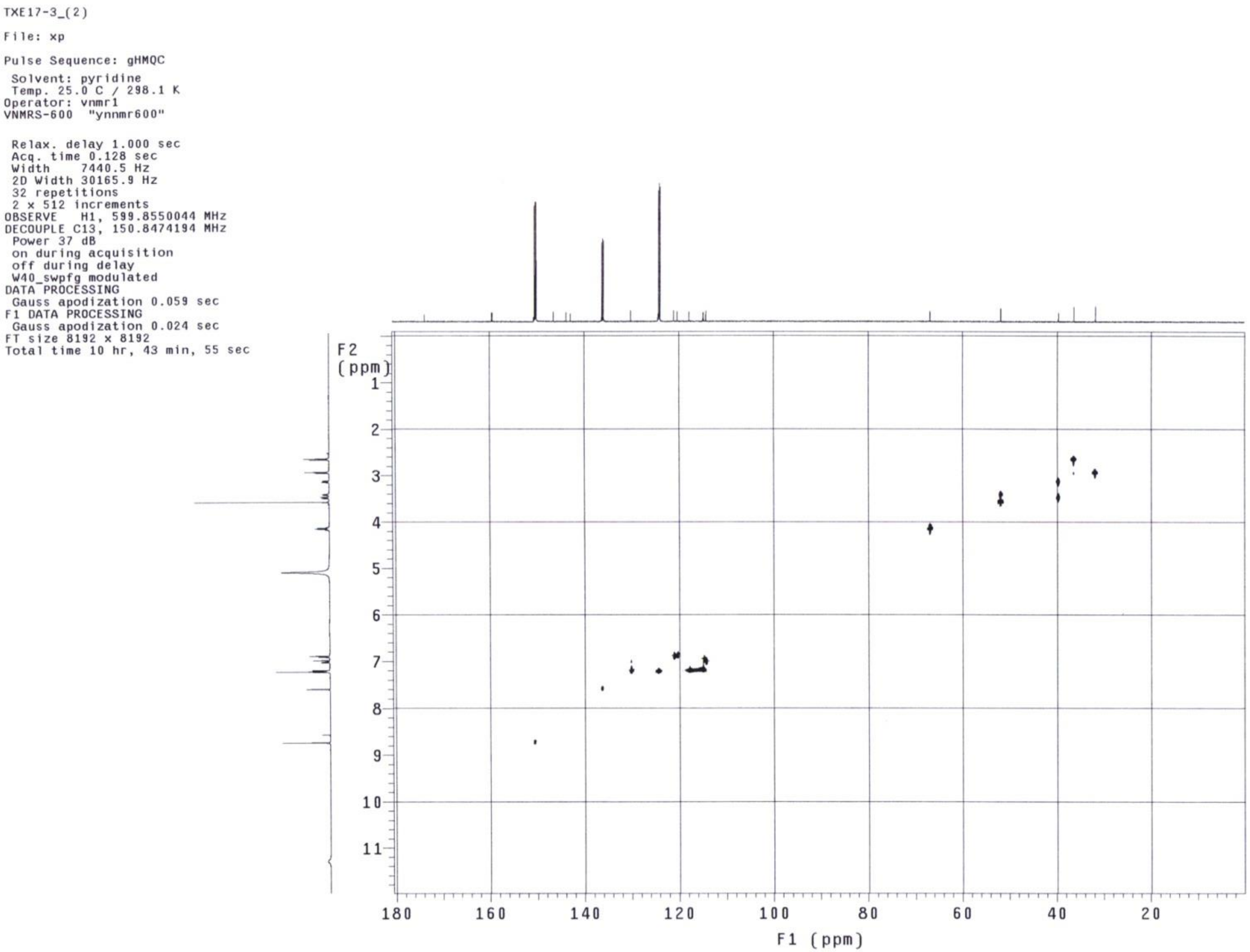

Figure S11: HMQC spectrum (pyridine-d_5, 600 MHz) of compound **2.**

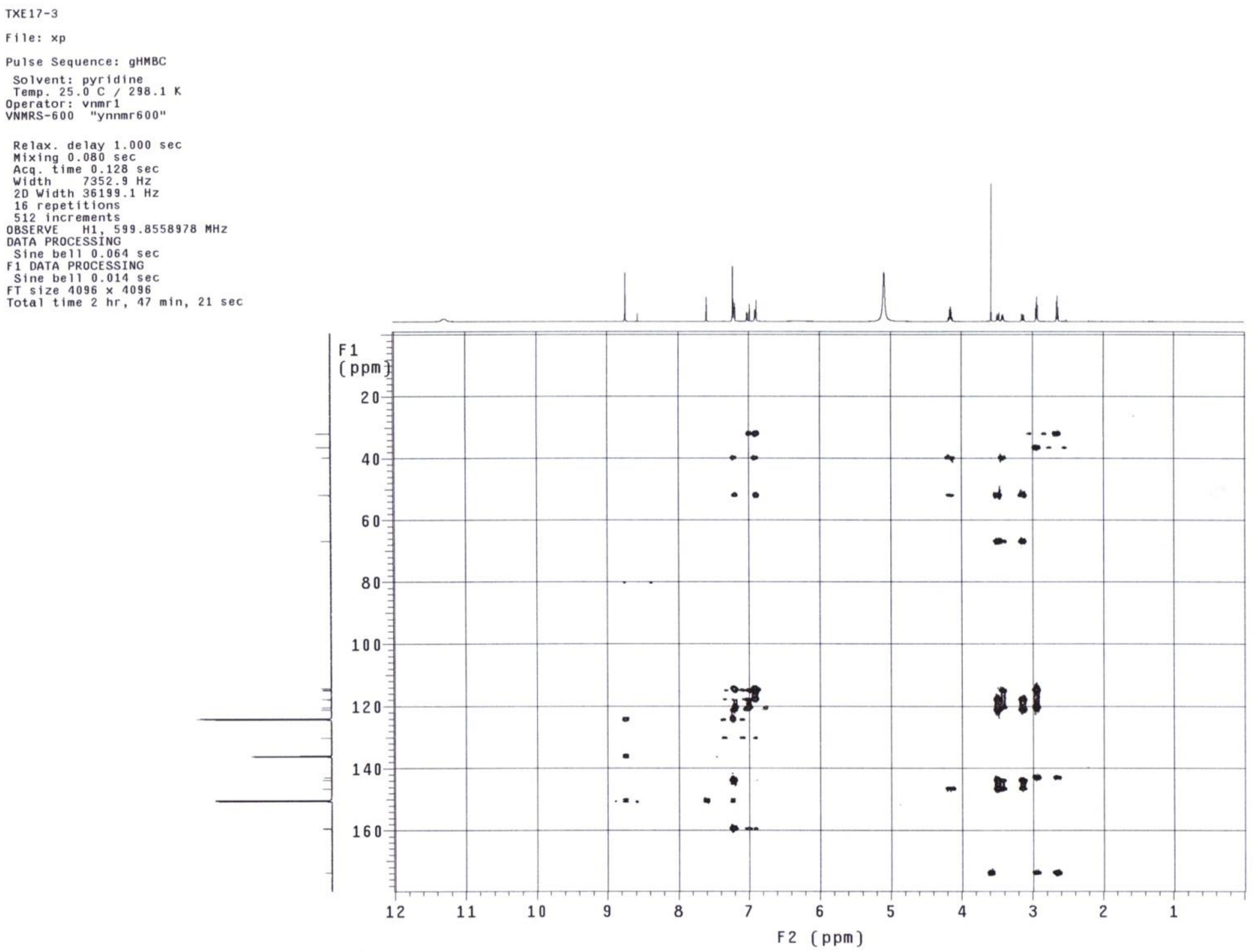

Figure S12: HMBC spectrum (pyridine-d_5, 600 MHz) of compound **2.**

File: 10220
Sample: TXE-2

Date Run: 12-12-2013
Ionization mode: EI+

Time Run: 09:56:10

Instrument: JEOL JMS600
Inlet: Direct Probe

R.T.: 2:47.3

Scan: 140
Base: m/z 330; 8.4%FS

TIC: 684936

Selected Isotopes : $C_{0-50}H_{0-100}O_{0-10}$

Error Limit : 20 ppm

Measured Mass	***% Base***	***Formula***	***Calculated Mass***	***Error***
330.1469	100.0%	$C_{26}H_{18}$	330.1409	-18.0
		$C_{19}H_{22}O_5$	330.1467	-0.6
		$C_{12}H_{26}O_{10}$	330.1526	17.0

Figure S13: HREIMS of compound **2.**

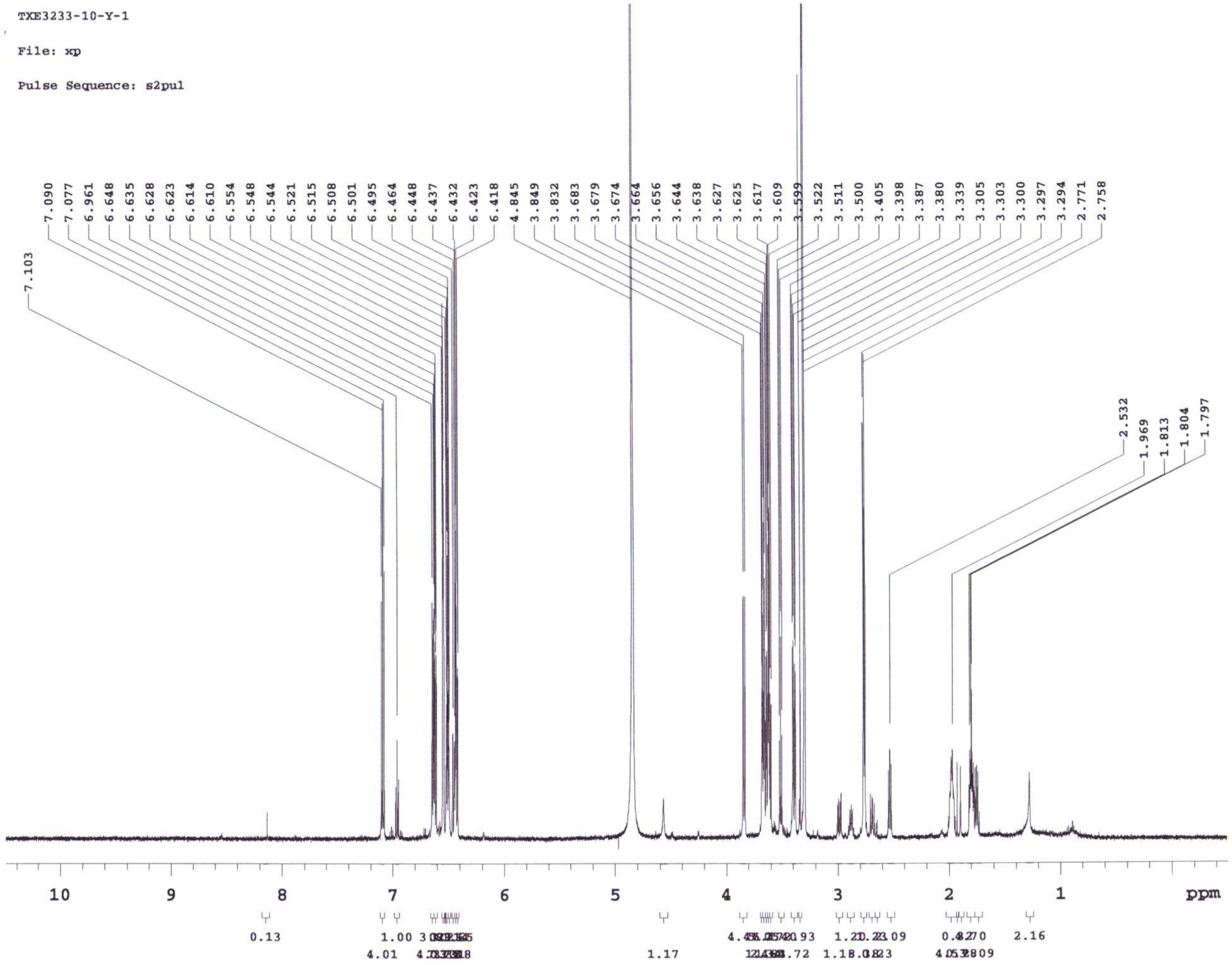

Figure S14: ^{1}H NMR spectrum (CD_3OD, 600 MHz) of compound **3.**

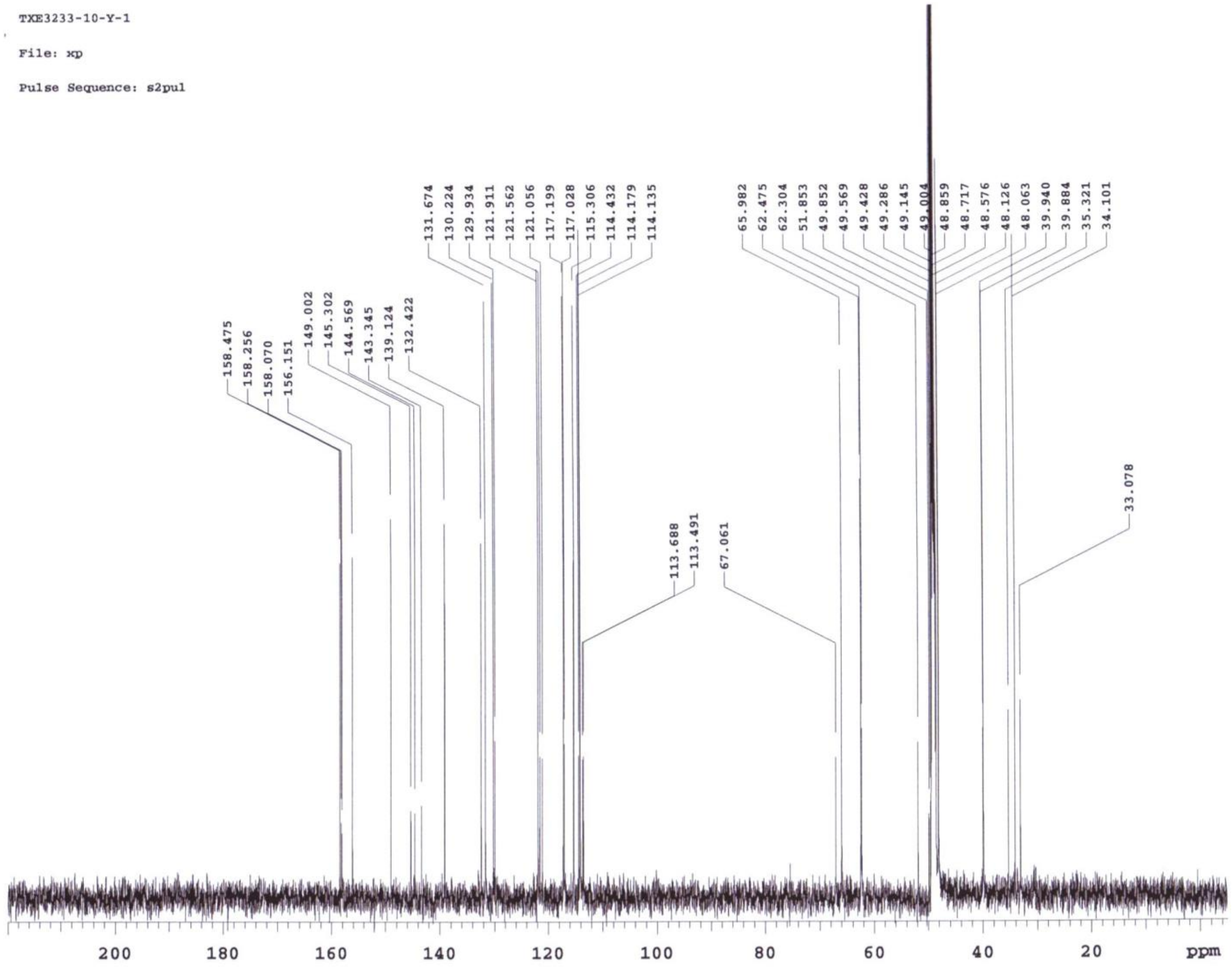

Figure S15: ^{13}C NMR spectrum (CD_3OD, 600 MHz) of compound **3.**

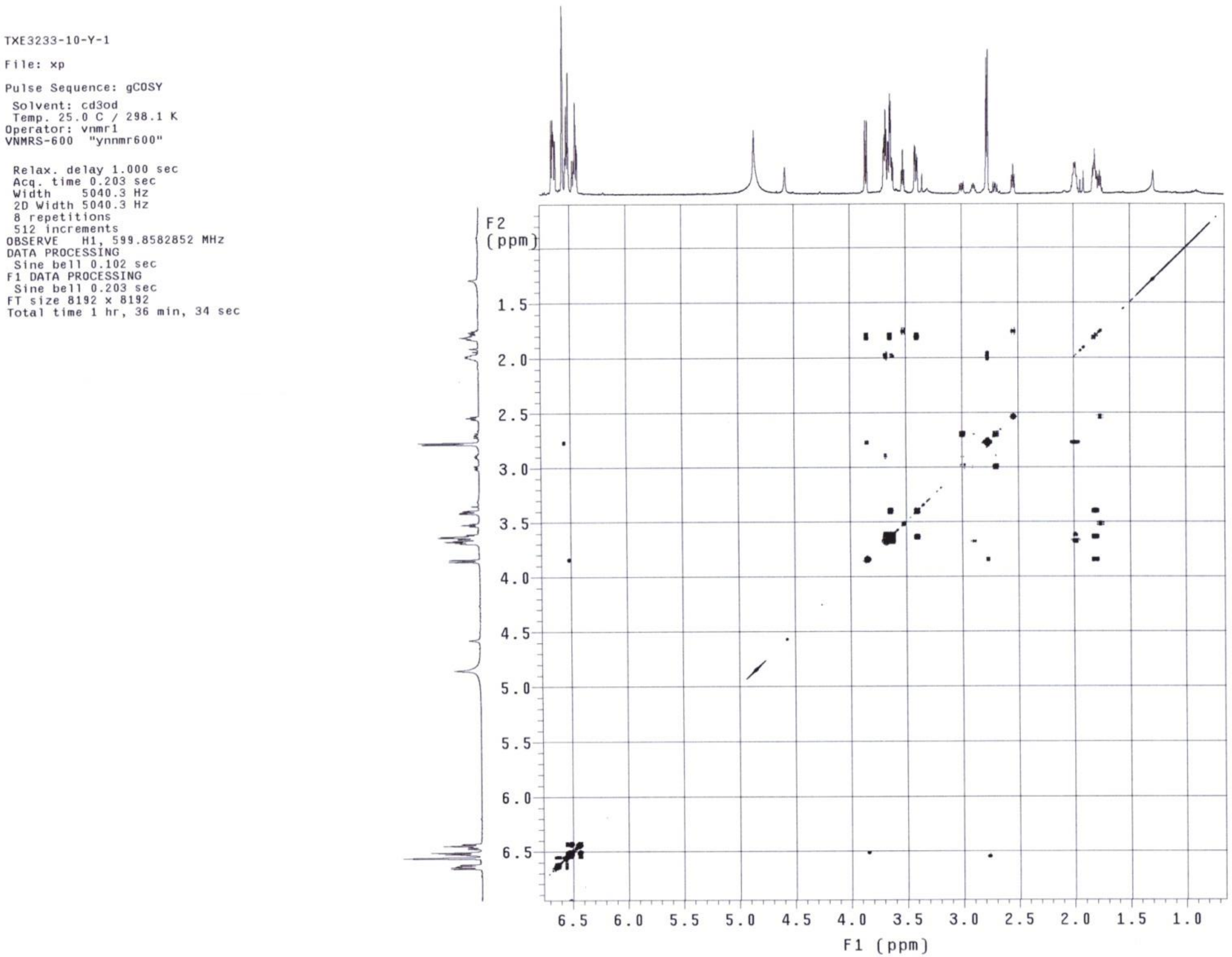

Figure S16: $^{1}H,^{1}H$ COSY spectrum (CD_3OD, 600 MHz) of compound **3.**

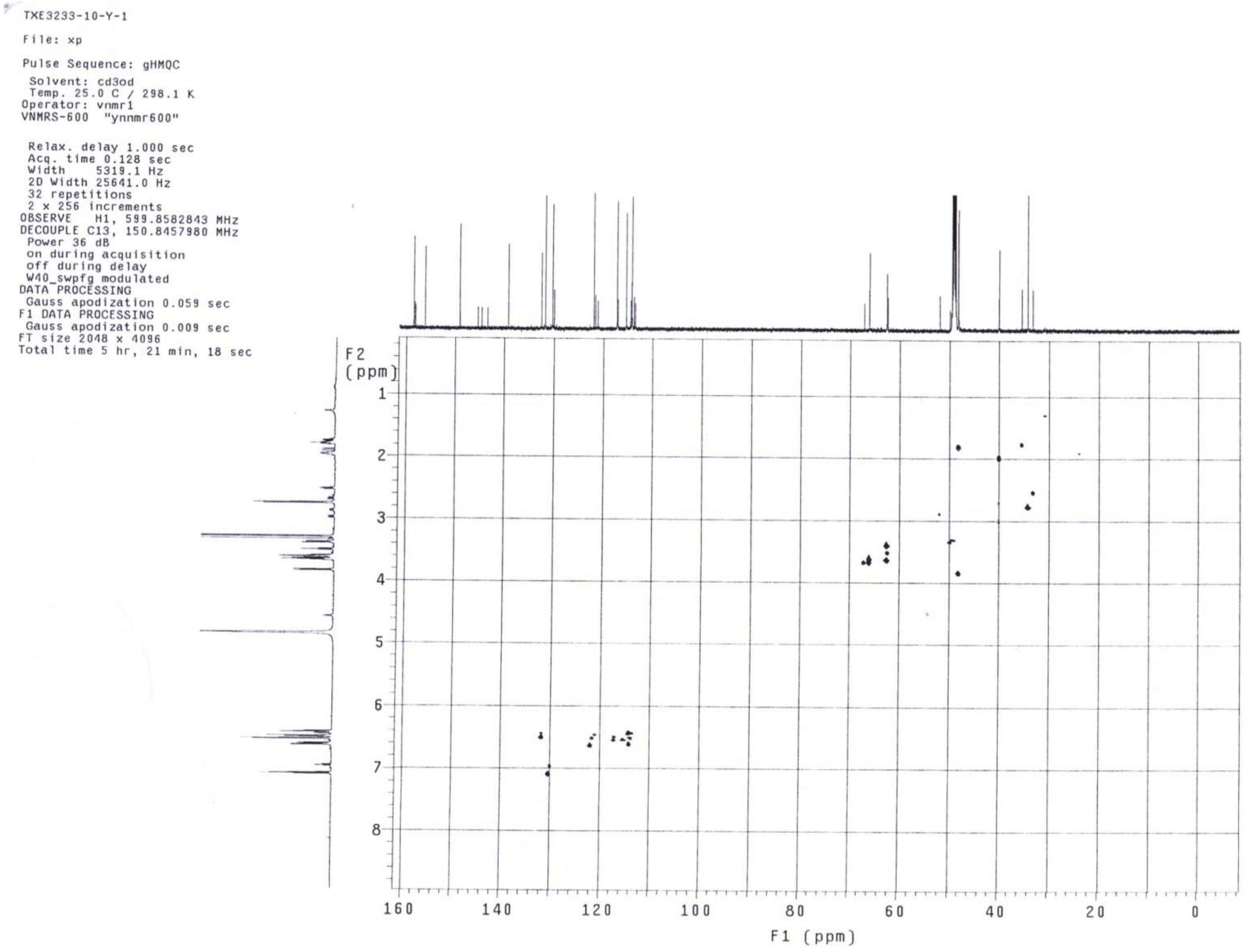

Figure S17: HMQC spectrum (CD_3OD, 600 MHz) of compound **3.**

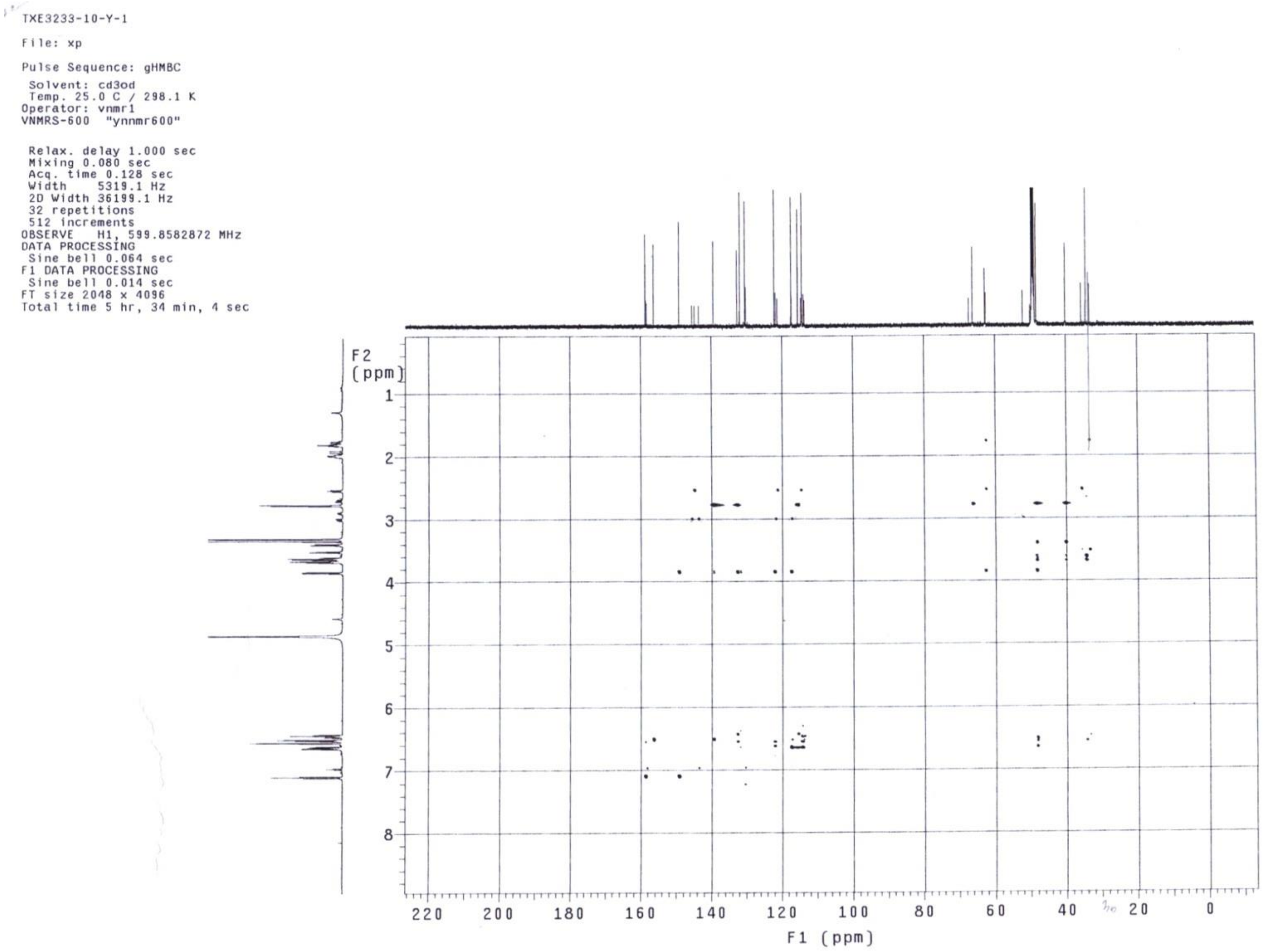

Figure S18: HMBC spectrum (CD_3OD, 600 MHz) of compound **3.**

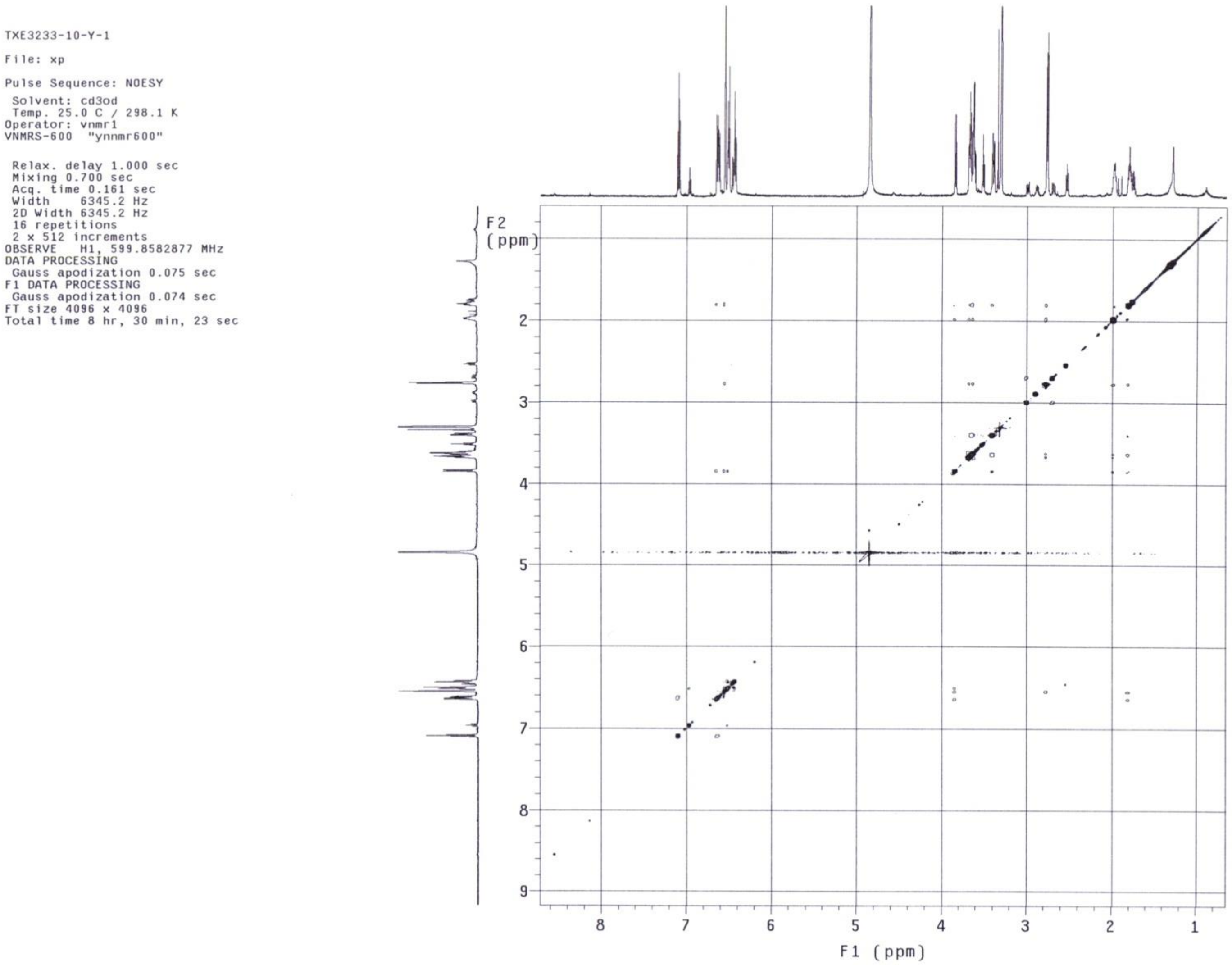

Figure S19: NOESY spectrum (CD_3OD, 600 MHz) of compound **3.**

```
[ Elemental Composition ]                                         Page: 1
Data   : EI636                       Date : 01-Apr-2014 20:03
Sample: TXE 3
Note  : -
Inlet : Direct                       Ion Mode : EI+
RT : 0.31 min                        Scan#: (14,16)
Elements : C 40/0, H 49/0, O 10/0
Mass Tolerance       : 1000ppm, 3mmu if m/z < 3, 5mmu if m/z > 5
Unsaturation (U.S.) : -0.5 - 10.0

Observed m/z Int%   Err[ppm / mmu]    U.S.  Composition
  300.1360   100.0      -0.7 /  -0.2    9.0  C 18 H 20 O 4
```

```
[ Theoretical Ion Distribution ]                                  Page: 1
Molecular Formula : C18 H20 O4
                       (m/z   300.1362, MW   300.3544, U.S.  9.0)
Base Peak :    300.1362, Averaged MW :    300.3524(a),    300.3532(w)

    m/z       INT.
  300.1362 100.0000 ******************************************************
  301.1395  20.1726 ************
  302.1422   2.7252 **
  303.1448   0.2766
  304.1474   0.0226
  305.1500   0.0016
```

Figure S20: HREIMS of compound **3.**
